# Icariin shapes post-withdrawal fecal resistome dynamics in layer hens

**DOI:** 10.1186/s40104-026-01433-8

**Published:** 2026-06-04

**Authors:** Jiaqi Zhang, Xiaohan Shi, Siyi Peng, Chi Zhang, Shiyan Qiao, Haitao Yu

**Affiliations:** 1 Hubei Key Laboratory of Animal Nutrition and Feed Science, Wuhan Polytechnic University, Wuhan, 430023 China; 2 State Key Laboratory of Animal Nutrition and Feeding, College of Animal Science and Technology, China Agricultural University, No. 2 Yuanmingyuan West Road, Haidian District, Beijing, 100193 China

**Keywords:** Antibiotic resistance genes, Co‑selection, Icariin, Layer hen, Mobile genetic elements

## Abstract

**Background:**

While the livestock industry actively seeks alternatives to antibiotics, residual low-dose exposures continue to drive the spread of antibiotic resistance genes (ARGs). Icariin, a plant-derived compound, is recognized for improving poultry growth and immunity. However, it remains unclear how this compound influences the environmental persistence of ARGs, mobile genetic elements (MGEs), and horizontal gene transfer (HGT) during the vulnerable recovery phase after antibiotic withdrawal.

**Results:**

We designed a two-phase feeding trial with laying hens, using longitudinal metagenomic sequencing to track post-withdrawal resistance dynamics. Following initial exposure to a low-dose antibiotic mixture that established a baseline of elevated resistance, hens received either a basal diet, an icariin-supplemented diet, or a copper sulfate-supplemented diet. The data indicate that icariin supplementation consistently reduced the burdens of both ARGs and MGEs. It also suppressed the potential for HGT and restricted the diversity of microbial hosts harboring these resistance elements. Conversely, copper sulfate—a traditional metal-based additive—exacerbated resistance risks by expanding both the abundance and the host range of ARGs and MGEs. Across all treatments, the population of *Escherichia* and the prevalent ARG subtype *bacA* correlated strongly with total resistance loads, tracking the overall resistome burden.

**Conclusions:**

Compared to conventional copper sulfate treatments, icariin facilitates a safer ecological recovery in the poultry gut by actively lowering ARG and MGE reservoirs after antibiotic withdrawal. These genomic insights, combined with its known physiological benefits, support icariin as a sustainable feed additive. Furthermore, the *Escherichia*-*bacA* correlation provides a reliable, streamlined indicator for monitoring resistance risks in farm environments. However, as these findings rely on short-term fecal metagenomic tracking, further validation through multi-environment studies is warranted.

**Graphical Abstract:**

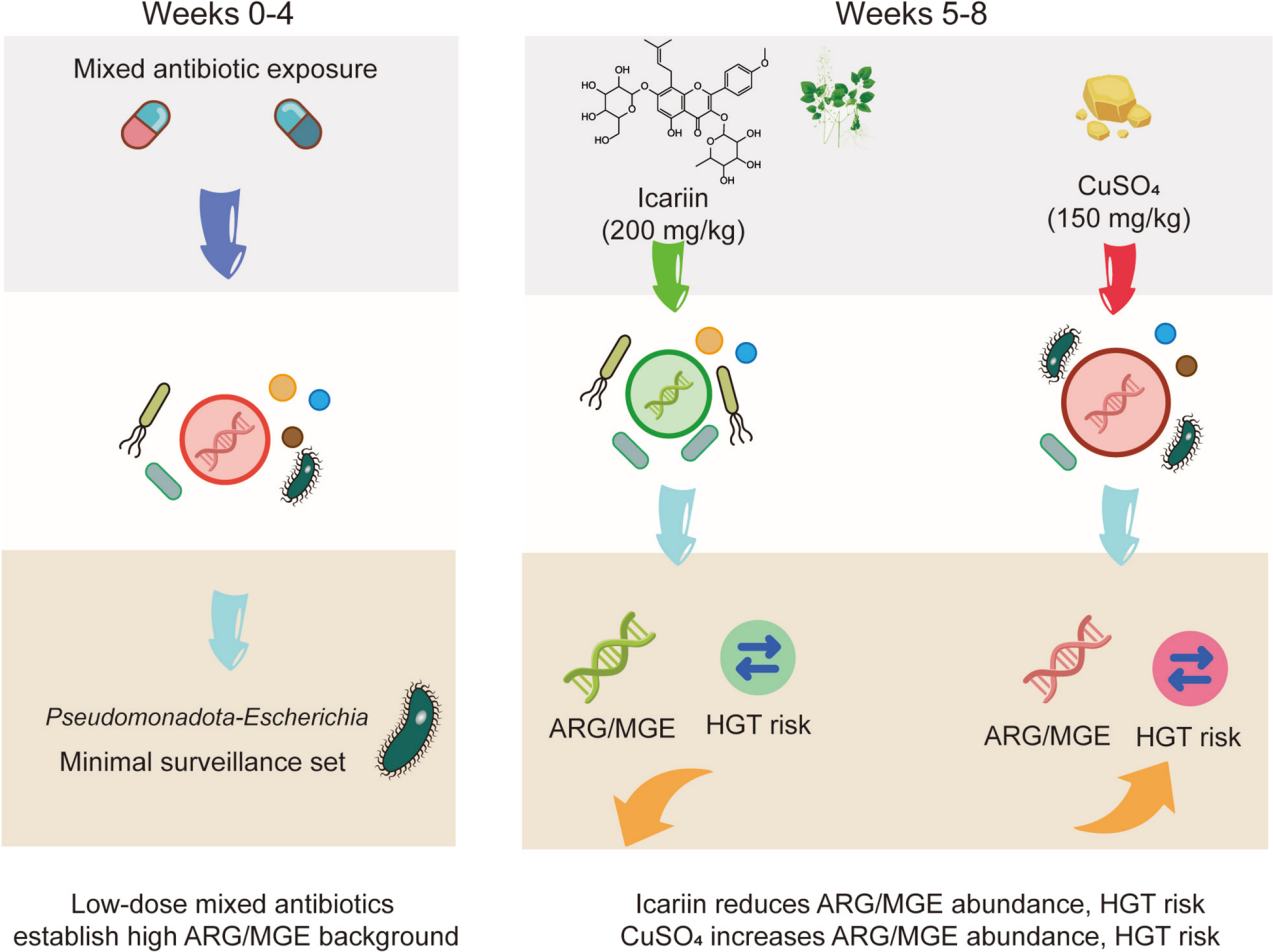

**Supplementary Information:**

The online version contains supplementary material available at 10.1186/s40104-026-01433-8.

## Introduction

ARGs originating from livestock and poultry production continue to present a structural challenge to global public health [1–3]. Even within modern antibiotic-free (AF) farming systems, extensive pools of ARGs persist across soil, water, and air environments [4–6]. The animal gut microbiome acts as a central exchange hub, where HGT facilitates the movement of ARGs between commensal bacteria and environmental pathogens. This continuous genetic exchange stabilizes mobile resistance elements and establishes persistent contamination chains that extend into surrounding ecosystems [7–9]. Consequently, the development of feed additives must prioritize dual objectives: securing animal growth and immunity while actively mitigating the environmental footprint of resistance elements [10, 11].

Currently, various antibiotic alternatives have emerged in livestock and poultry farming. Among them, plant-derived phytochemicals and metal-based feed additives have attracted considerable attention. These growth promoters exhibit unique properties in improving growth performance, gut health, immune status, or exerting subinhibitory antimicrobial effects [12–14]. Metal-based additives, such as copper sulfate (CuSO_4_), are widely used for their growth-promoting and subinhibitory antimicrobial properties. However, sustained heavy metal exposure often triggers co-selection mechanisms, potentially maintaining or even amplifying ARG networks long after clinical antibiotics have been withdrawn [15–17]. In contrast, plant-derived phytochemicals offer a different ecological profile. Our previous research identified icariin—a prenylated flavonoid glycoside extracted from the genus *Epimedium*—as a physiologically beneficial feed additive that enhances intestinal barrier function and modulates the avian gut microbiota [18, 19]. Although icariin effectively suppresses opportunistic pathogens while promoting beneficial taxa, its direct capacity to mitigate the burdens of ARGs and MGEs, and to reduce HGT risks, during the vulnerable post-antibiotic recovery phase has not been systematically evaluated.

Therefore, expanding beyond the traditional evaluation of feed additives, we conducted a two-phase dynamic monitoring experiment in laying hens that closely reflects the actual post-antibiotic conditions in poultry farming. In the initial phase (weeks 0 to 4), all hens received low doses of a mixture of antibiotics in their feed to establish a consistent and high-level ARG abundance in their feces. In the second (withdrawal) phase (weeks 5 to 8), hens were randomly assigned to three groups: a basal diet group, an icariin group, and a CuSO_4_ group (as a positive control). Subsequently, we conducted continuous monitoring of fecal ARGs, MGEs, host ranges, and putative HGT risks using a unified metagenomics workflow. Tracking these resistance patterns during the critical withdrawal period reveals how different dietary interventions influence the environmental persistence of resistance genes over time. Within this microbial context, we aimed to demonstrate that plant-derived icariin possesses a distinct capability to suppress pathogen-associated resistance genes, contrasting with the co-selection effects typically observed with traditional metal additives like copper sulfate. Finally, to translate these high-dimensional metagenomic findings into routine farm management, we further explored whether the complex resistance network could be summarized into a practical surveillance target based on the *Escherichia*-*bacA* association, thereby providing an accessible approach for environmental resistance monitoring.

## Materials and methods

### Animals and diets

Forty‑five 310‑day‑old laying hens were obtained from a single commercial farm with no recorded history of antimicrobial use. Birds were housed individually in a stacked‑cage system in three environmentally controlled rooms (20–24 °C, 50%–65% relative humidity, 16L:8D photoperiod) with ad libitum access to feed and water. Manure boards were cleaned daily. Throughout the experiment, the hens were fed a standard basal diet formulated to meet their nutritional requirements. The detailed ingredient composition and nutrient levels of this basal diet are provided in Table S1. After arrival, hens underwent a 28‑day acclimation period to stabilise husbandry conditions and microbiota baselines.

The components of the antibiotic mixture were obtained as follows: chlortetracycline (Shanghai Yuanye Biotechnology Co., Ltd., Shanghai, China; USP grade, 900 μg/mg; Cat. No. S17011-100 g), sulfamethoxazole (Shanghai Yuanye Biotechnology Co., Ltd., Shanghai, China; BR grade, 99% purity; Cat. No. S17084-100 g), and penicillin G potassium salt (Aladdin Biochemical Technology Co., Ltd., Shanghai, China; ≥ 98%; Cat. No. P102194-100 g).

**Fig. 1:**
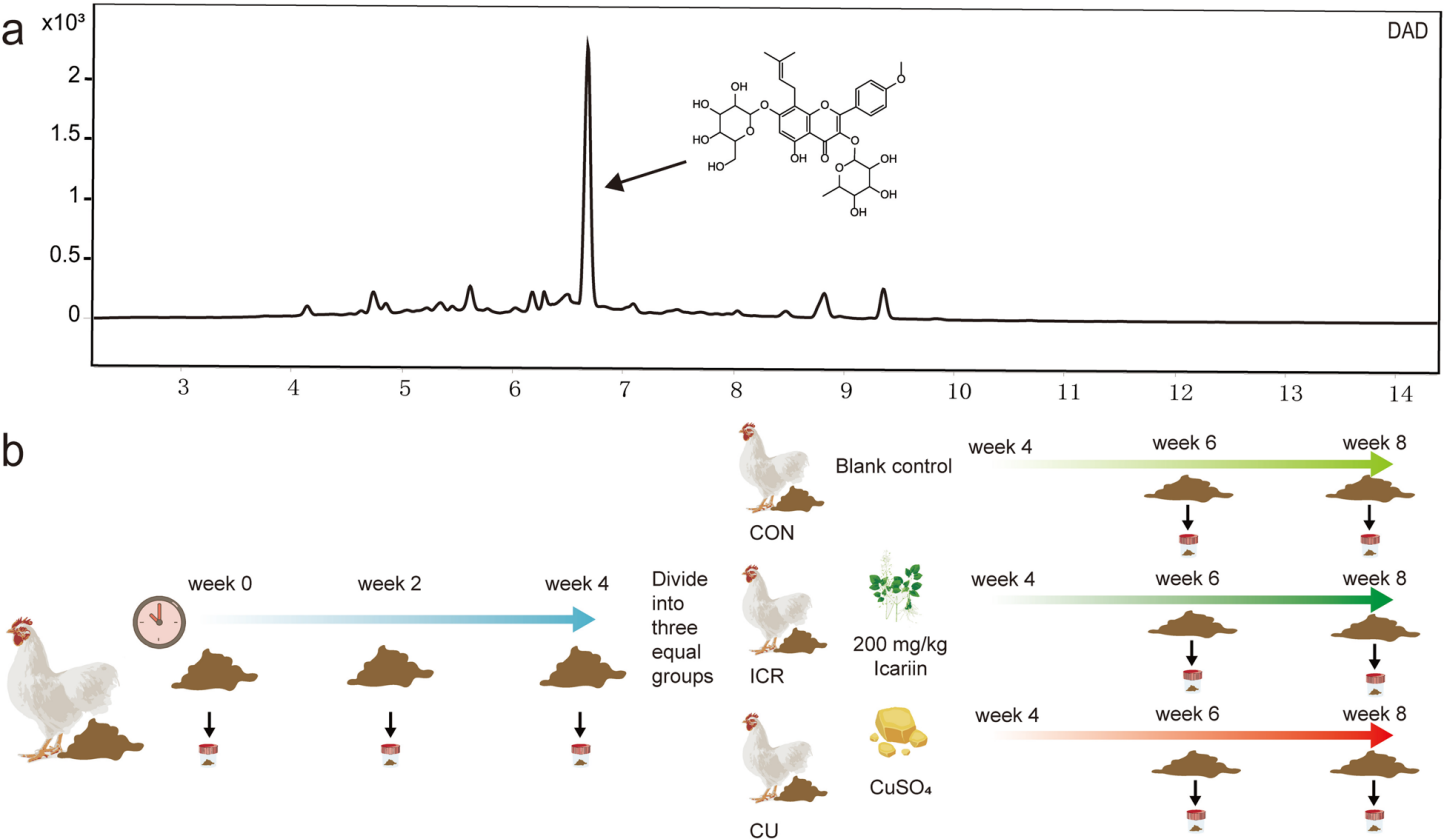
Chromatographic profile of icariin and experimental design. **a** Chemical structure and high-performance liquid chromatography (HPLC) chromatogram of icariin. **b** Schematic representation of the experimental animal grouping, treatment strategies, and fecal sampling timeline in layer hens

Icariin was obtained from Shanghai Yuanye Biotechnology Co., Ltd. (HPLC purity ≥ 98%, Cat. B21576-100 mg) and was supplied as a crystalline powder. Purity information was provided by the manufacturer (certificate of analysis), and the compound was additionally checked by in-house HPLC (Fig. 1a). The copper additive was copper(II) sulfate pentahydrate purchased from Shanghai Yuanye Biotechnology Co., Ltd. (AR grade, ≥ 99%, Cat. S24261-500 g). The copper inclusion level was set at 150 mg/kg of elemental Cu (approximately 590 mg/kg CuSO_4_·5H_2_O). Because copper use in poultry diets is regulated and practice-dependent, we selected 150 mg/kg elemental Cu as a field-relevant pharmacological dose (commonly reported within 125–250 mg/kg in commercial settings), while noting that lower limits have been adopted in some regions such as the European Union [20–22]. This dose was used as a positive-control pressure to compare resistome trajectories under a conventional metal-based additive versus a phytochemical intervention.

### Experimental design

The animal trial was carried out in two sequential phases (Fig. 1b). In phase 1 (antibiotic exposure weeks 0–4, establish a comparable post-exposure baseline across birds), all 45 hens were given the identical low-dose in-feed antibiotic blend (ASP250) for 4 consecutive weeks. The mixture contained chlortetracycline, sulfamethoxazole and penicillin at concentrations of 100, 100 and 50 g/ton of feed, respectively, adhering to the protocol outlined by Looft et al. [23].

In phase 2 (from week 4 to week 8), the administration of antibiotics was discontinued. The hens were stratified according to their body weight and laying performance, and randomly allocated into 3 groups: CON group, basal diet; ICR group, fed the basal diet supplemented with 200 mg/kg of icariin (ICR) and CU group, which was provided with a basal diet supplemented with 150 mg/kg of CuSO_4_ (CU). These diets were continued for an additional four weeks. Throughout the study, there were no recorded mortalities or adverse events associated with the interventions.

To ensure statistical rigor and prevent pseudoreplication during longitudinal tracking, all 45 hens were individually tagged. Within any specific sampling time point, the 15 individual birds in each treatment group served as independent biological replicates for cross-sectional comparisons. For temporal assessments bridging multiple weeks, samples obtained from the same hen were recognized as repeated measures. Accordingly, downstream analyses treated bird identity as the unit of repeated measurement.

All procedures were approved by the Animal Welfare and Ethics Committee of Wuhan Polytechnic University (approval No. WPU202410010) and complied with institutional and national guidelines for the care and use of laboratory animals.

### Fecal sampling

Fresh fecal samples were collected from all hens at weeks 0, 2 and 4 (during antibiotic exposure) and at weeks 6 and 8 (during the growth‑promoter phase). Sampling was performed within a fixed 2‑h window in the morning. Individual freshly voided droppings were aseptically collected from the trays beneath each cage using sterile disposable spatulas, avoiding contact with bedding or cage surfaces, placed into sterile microcentrifuge tubes, immediately snap‑frozen in liquid nitrogen or transported on dry ice, and stored at −80 °C until DNA extraction.

### DNA extraction and metagenomic sequencing

Microbial genomic DNA was extracted from chicken fecal samples using the E.Z.N.A.® Stool DNA Kit (Omega Bio-Tek, Norcross, GA, USA) according to the manufacturer's instructions. The concentration and purity of the extracted DNA were assessed using a NanoDrop 2000 spectrophotometer (Thermo Fisher Scientific, Wilmington, DE, USA). Metagenomic shotgun sequencing libraries were constructed and sequenced by Biozeron Biotechnology Co., Ltd. (Shanghai, China). For each sample, 1.0 μg of genomic DNA was randomly fragmented to an average size of approximately 450 bp using a Covaris S220 focused ultrasonicator (Covaris, Woburn, MA, USA), and sequencing libraries were subsequently prepared using end repair, A-tailing, adapter ligation, and limited-cycle PCR amplification. All libraries were sequenced on the Illumina HiSeq X platform (Illumina, San Diego, CA, USA) using a paired-end 150 bp (PE150) sequencing strategy with standard Illumina chemistry.

### Bioinformatics analysis

#### Quality control and filtering of clean reads

Raw sequencing reads were quality-filtered using Trimmomatic v0.36 [24] (https://github.com/usadellab/Trimmomatic) to remove adapter sequences, contaminants, and low-quality reads. The filtering parameters used were ILLUMINACLIP:adapters.fa:2:30:10, SLIDINGWINDOW:4:15, and MINLEN:75. To eliminate host-derived reads, clean reads were aligned to the *Gallus gallus* reference genome using BWA-MEM [25] with parameters as follows: -M -k 32 -t 16 (https://bio-bwa.sourceforge.net/bwa.shtml). Reads mapping to the host genome were removed from subsequent analysis. After removing host contamination and low-quality data, the high-quality clean reads were de novo assembled into contigs using MEGAHIT [26] with the parameter --min-contig-len 500 and default k‑mer lists.

#### Metagenomic binning and assembly

Metagenomic binning was performed using MetaBAT2 v2.12.1 [27] and MaxBin2 v2.2.7 [28], which clustered contigs into metagenome-assembled genomes (MAGs) based on sequence composition and read coverage information. To improve binning accuracy, clean reads were mapped back to the contigs using BWA-MEM (v0.7.17, https://bio-bwa.sourceforge.net) with default parameters, and the coverage estimation for each contig was performed using SAMtools v1.9 (http://www.htslib.org/) [29]. The sequencing depth of each contig was used for subsequent binning. For MetaBAT2, the default parameters were used, with a minimum contig length set to 1,500 bp (--minContig 1500). MaxBin2 was run with the default probabilistic model and a minimum contig length of 1,500 bp. The binning results from MetaBAT2 and MaxBin2 were subsequently refined with DAS Tool v1.1.2 [30] to integrate and optimize bin assignments, thereby producing non-redundant, higher‑quality MAGs.

#### Quality assessment and taxonomic annotation of MAGs

The completeness and contamination of each MAG were assessed with CheckM v1.2.1 [31], which uses lineage-specific single-copy marker genes for quality evaluation and assigns taxonomic lineages based on phylogenetic placement. MAGs with completeness ≥ 50% and contamination ≤ 10% were retained for further analysis as medium-quality draft genomes. The final taxonomic assignments for each MAG were obtained using the Genome Taxonomy Database Toolkit (GTDB-Tk, version r214, https://gtdb.ecogenomic.org/).

#### Functional annotation of ARGs, MGE, MRG, and virulence factors

To identify functional genes, open reading frames (ORFs) were predicted using Prodigal v2.6.3 [32]. The resulting protein sequences were aligned to curated reference databases using DIAMOND BLASTP [33]. These databases included the Structured Antibiotic Resistance Gene (SARG) database for ARGs [34], Mobile Genetic Elements Database for MGEs [35], BacMet database for metal resistance genes (MRGs) [36], and Virulence Factor Database (VFDB) [37].

The alignment results were filtered using the following thresholds: sequence identity ≥ 60% and query coverage against the reference ≥ 60%. To quantify the relative abundance of the functional genes, clean reads were mapped back to the assembled contigs to determine the read counts assigned to each annotated open reading frame. For cross-sample comparability, the raw mapped read counts were mathematically normalized to correct for variations in both target gene length and sequencing depth, reported as ppm (length-adjusted reads per million total reads). The calculation was performed using the following formula:

$${ppm}_{i}=\frac{{C}_{i}/{L}_{i}}{N}\times {10}^{6}$$

where *C*_*i*_ is the number of mapped reads assigned to the specific gene/element *i*, L_*i*_ is the sequence length of the target, and *N* is the total number of high-quality clean reads in the specific sample. The total abundance of ARGs or MGEs per sample was calculated by summing the ppm values of all detected corresponding genes/elements. Only hits that met these criteria were selected for subsequent analyses.

To prioritize genomes with increased genetic risk potential, MAGs annotated with ˃10 total functional genes (ARGs, MGEs, MRGs, or VFDB genes) were defined as high-risk genomes. To explore host attribution and gene mobility, a randomly subsampled 1.0 GB subset of clean reads was analyzed using BPTracer (https://github.com/LorMeBioAI/BP-Tracer). BPTracer was used to trace potential source hosts and classify types and subtypes of ARG/MGE, with parameters set as: minimum alignment length = 25 amino acids, similarity ≥ 95%, and E-value ≤ 1 × 10^−5^. The resulting functional gene–host classification matrix was used to reconcile annotations in cases where multiple plausible hosts were inferred.

#### HGT analysis based on MAGs

To investigate HGT, contigs ≥ 2 kb from MAGs were analyzed using WAAFLE (https://github.com/biobakery/waafle) as implemented within the BP‑Tracer workflow. HGT detection employed the JGT algorithm, which consisted of (1) nucleotide-level alignment of contigs against a reference pangenome using waafle_search.py, and (2) taxonomic assignment and HGT inference via waafle_orgscorer.py. The HGT frequency was normalized according to the ppm abundance (parts per million sequences) of each MAG, based on the method described by Hsu et al. [38].

#### Pathogen analysis

Pathogen identification at the MAG level was conducted based on a curated list of pathogenic bacterial species compiled by Yi et al. [39]. MAGs meeting quality thresholds (completeness ≥ 50% and contamination ≤ 10%) were taxonomically classified by CheckM/GTDB‑Tk. MAGs assigned to the pathogenic species listed in the reference study were designated as putative pathogenic MAGs. Their distributions and abundances in the samples were analyzed to evaluate potential health risks. All sequencing data generated in this study have been deposited in the NCBI Sequence Read Archive under BioProject accession number PRJNA1345554.

### Data processing and statistical analysis

All statistical analyses and visualizations were performed in R (v4.1.2 unless otherwise specified; https://www.r-project.org). Community structural differences were characterized using principal coordinates analysis (PCoA) based on Bray–Curtis distances, with each point representing an individual sample and color-coded by time point. The differential abundance of the microbiome, ARGs, and MGEs was assessed using DESeq2, and significance thresholds of |log_2_(fold change)|> 1 and *P* < 0.05 were applied to identify taxa and genes exhibiting significant shifts. The correlation structure was evaluated by computing Spearman rank correlations among the microbiome, ARG, and MGE abundances using the ggpubr stat_cor function, as well as Pearson correlation coefficients to examine linear relationships. Group-wise hypothesis testing typically employed a one-way ANOVA, and pairwise contrasts were conducted using Tukey’s post hoc multiple-comparison procedure through the agricolae package; the same workflow was used to compare α-diversity indices. Statistical comparisons of HGT frequency were performed in R (v4.2); non-parametric tests (Kruskal–Wallis) were conducted when normality assumptions were not met. Pairwise comparisons were conducted using Dunn’s test with Benjamini–Hochberg correction for multiple testing, and counts and frequencies were summarized accordingly. Sankey diagrams were generated with the sankeyD3 package to show directional flows and relative contributions among components.

To capture time-resolved signals linked to post-withdrawal trajectories, we ranked ARG subtypes by their overall mean ppm abundance across weeks 4–8 and selected the top 10 most abundant subtypes for each treatment. Within each treatment (ICR, CU), trend consistency of each subtype was assessed by correlating subtype abundance with sampling week treated as an ordinal variable (4, 6, 8) using Spearman’s ρ; a subtype was deemed “trend‑consistent” if the correlation direction matched the group‑level direction of total ARG ppm (ICR decreasing; CU increasing) and the association reached FDR-adjusted significance (Benjamini–Hochberg, *q* < 0.05). The two trend‑consistent subtype sets (ICR and CU) were compared using Venn analysis (R packages VennDiagram or ggvenn) to identify shared markers.

Shared ARG subtypes were mapped to putative hosts using the BP-Tracer pipeline (default settings unless otherwise stated) to obtain genus-level host assignments. Pairwise associations between candidate host genera and shared ARG subtypes were quantified with Spearman rank correlations across samples; multiple testing was controlled by Benjamini–Hochberg correction, and edges were retained if ρ > 0.6 and *q* < 0.05. A bipartite network was constructed and visualized with igraph and ggraph, and node degree was used to summarize hubness at the gene and genus levels.

Analytical approaches were carefully selected to accommodate the longitudinal sampling design. When comparing differences among the basal diet, icariin, and copper sulfate groups at a single distinct time point, standard independent tests were applied using the 15 biological replicates per group. Conversely, to evaluate dynamic microbiome and resistome shifts within the same group across the experimental timeline, the non-independence of sequential samples was explicitly addressed. Individual bird identification was incorporated as a blocking factor in permutational multivariate analyses of variance and treated as a random effect in mixed-effects models. This design-aware handling of repeated measures was used to separate within-bird temporal change from between-bird variation. Regarding data visualization, for all stacked-bar plot visualizations throughout the study (including compositional profiles of microbial taxa, ARGs, MGEs, putative hosts, and HGT frequencies), each bar represents an individual biological replicate (one individual animal, *n* = 3 per group at each time point) rather than a group average, thereby illustrating intra-group consistency.

## Results

### Temporal dynamics of the fecal microbiome

**Fig. 2:**
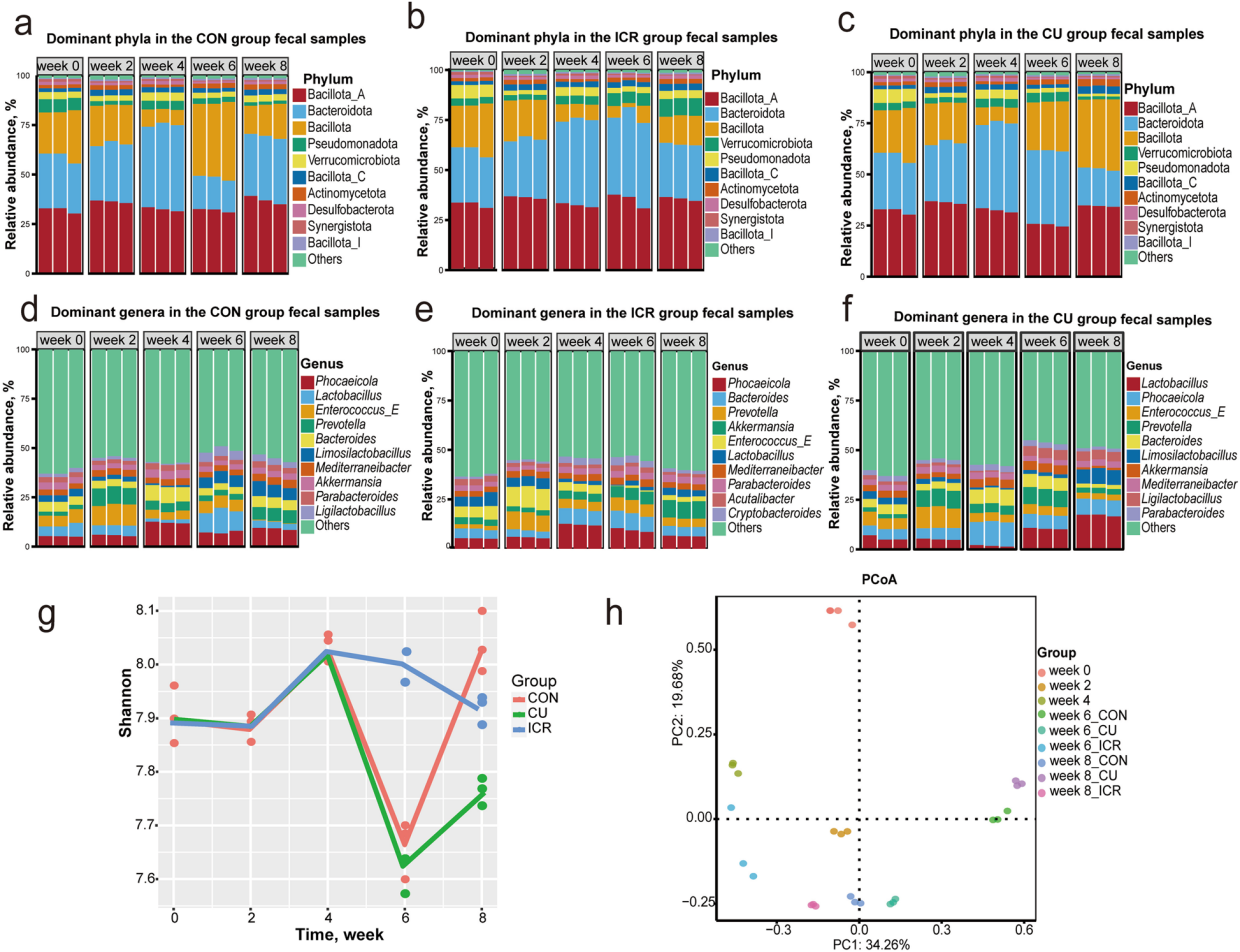
Temporal dynamics of the fecal microbiome in layer hens under different growth-promotion strategies. **a**–**c** Stacked bar plots of phylum-level composition (%) at each time point for the CON, ICR, and CU groups, respectively. **d**–**f** Stacked bar plots of genus-level composition (%) for the three groups. **g** Line chart of Shannon index across time points. **h** Principal coordinates analysis (PCoA) scatter plot based on Bray–Curtis distances depicting overall microbial community structure. In the stacked-bar plots (**a**–**f**), each bar represents one individual fecal sample (biological replicate), with three replicates (*n* = 3) shown for each group at each time point

To characterize the temporal shifts in the fecal microbiome, longitudinal metagenomic profiling was conducted according to a predefined schedule (Fig. 2). Following a four-week initial exposure to a low-dose antibiotic mixture (ASP250) designed to establish a unified baseline of resistance, laying hens were randomly allocated to three post-withdrawal dietary interventions: a basal diet (CON), an icariin-supplemented diet (ICR, 200 mg/kg), and a copper sulfate-supplemented diet (CU, 150 mg/kg), with monitoring continued until week 8. Throughout the experimental period, the gut microbiome retained a highly stable phylum-level core predominantly composed of Bacteroidota (mean relative abundance 34.80%), Bacillota_A (32.66%), and Bacillota (15.87%) (Fig. 2a–c). During the initial antibiotic exposure phase (weeks 0–4), the administration of bacteriostatic agents restructured early colonization niches without fundamentally displacing this core, enriching prevalent genera such as *Phocaeicola* (11.08%), *Lactobacillus* (5.34%), and *Enterococcus_E* (6.09%) (Fig. 2d–f). Upon antibiotic withdrawal (weeks 4–8), the compositional trajectories of the microbiota diverged substantially across treatments. The CON group exhibited a spontaneous ecological recovery characterized by the sustained dominance of *Phocaeicola*, *Lactobacillus*, and *Bacteroides*. In contrast, while the ICR group retained high abundances of *Phocaeicola* and *Bacteroides*, it was specifically distinguished by a notable proliferation of *Akkermansia* (reaching 6.80%). The CU group, conversely, became co-dominated by *Lactobacillus* and *Phocaeicola*, accompanied by an expansion of *Prevotella* to 4.50%.

Alpha diversity, as measured by the Shannon index, exhibited minor fluctuations during the early antibiotic exposure (from 7.92 at week 0 to 7.89 at week 2) before peaking at 8.06 by week 4. Notably, the cessation of antibiotic treatment triggered a marked ecological perturbation at week 6, where both the CON and CU groups experienced a sharp decline in Shannon diversity. This transient drop likely reflects an acute ecological response to the abrupt removal of bacteriostatic constraints, wherein fast-growing opportunistic taxa rapidly proliferated to occupy newly vacated niches, thereby compromising overall community evenness. Subsequently, the CON group demonstrated a natural, V-shaped ecological rebalancing, with diversity rebounding to 8.04 by week 8. Conversely, the continuous application of copper sulfate in the CU group imposed a secondary, metallic selective pressure that persistently suppressed diversity to lower levels (7.61 at week 6 and 7.76 at week 8). Significantly, icariin supplementation in the ICR group effectively mitigated this transitional turbulence, sustaining high community complexity (8.01 at week 6 and 7.92 at week 8) and demonstrating a distinct capacity to buffer environmental fluctuations and promote microbiome resilience during post-antibiotic recovery (Fig. 2g). Consistent with these findings, principal coordinates analysis (PCoA) based on Bray–Curtis dissimilarities revealed a progressive structural displacement from the week 0 baseline, culminating in distinct spatial clustering among treatments by week 8, wherein the ICR and CU communities diverged furthest from the CON group (Fig. 2h).

Further examination of the putative pathogenic subcommunity identified a species-level profile primarily driven by *Enterobacter hormaechei_C*, *Escherichia coli*, *Lactobacillus sp946579245*, *Limosilactobacillus sp9052114235*, and *Megamonas hypermegale* (Fig. S1a–c). As expected, the initial antibiotic administration rapidly suppressed the pathogen burden, with the total relative abundance of these putative pathogens contracting from 5.24% at week 0 to 1.26% and 1.56% at weeks 2 and 4, respectively. During the post-withdrawal phase, pathogen abundance remained consistently low in both the CON group (peaking at 1.79% at week 6 before dropping to 1.02% by week 8) and the ICR group (averaging 1.50% across weeks 4–8). In stark contrast, the CU group exhibited a pronounced pathogen rebound, reaching 3.59% by week 8, characterized by a dominance shift from *E. coli* to *Lactobacillus sp946579245* (3.20%). Analysis of pathogen alpha diversity further confirmed this structural shift: while diversity indices naturally recovered in the CON and ICR groups following withdrawal, they continued to deteriorate in the CU group (declining from 1.93 at week 4 to 1.14 at week 8), indicating the formation of a low-diversity, highly skewed pathogenic architecture under prolonged copper exposure (Fig. S1d). These pathogen-level structural divergences were corroborated by distinct temporal clustering in the PCoA (Fig. S1e), thereby laying the ecological groundwork for the subsequent host-resolved evaluations of resistome dissemination risks.

### Temporal dynamics of ARGs and MGEs

To elucidate the developmental trajectories of antibiotic resistance, the absolute loads (ppm), lineage breadth (subtype richness), and compositional structures of ARGs and MGEs were dynamically profiled across all treatments (Figs. S2–S4). At baseline (week 0), the intrinsic ARG repertoire was highly diverse and predominantly composed of multidrug, macrolide-lincosamide-streptogramin (MLS), and vancomycin resistance classes, with *bacA*, *arlR*, and *vanRI* identified as the most abundant subtypes (Fig. S2a; Fig. S3a)—a profile consistent with historical exposure and ubiquitous environmental seeding. Following the introduction of in-feed antibiotics (weeks 0–4), the aggregate ARG load maintained broad stability; however, subtype richness underwent a severe contraction, plummeting from 56 subtypes at week 0 to merely 9 by week 4 (Fig. S2d). This indicates a potent selective pressure that systematically eliminated peripheral resistance determinants while strongly selecting for a narrow, core resistance spectrum.

Following antibiotic cessation (weeks 4–8), ARG trajectories exhibited treatment-specific divergence. The CON group underwent a transient surge in resistance load at week 6, followed by a natural attenuation to below-baseline levels by week 8, without fundamentally altering its core ARG composition (Fig. S2a). Conversely, the ICR intervention drove a monotonic, sustained decrease in ARG burdens from week 4 through week 8 across all major classes and subtypes, successfully reducing absolute resistance risks while preserving the underlying ecological structure (Fig. S2b; Fig. S3b). In stark contrast, copper sulfate supplementation in the CU group not only elevated the total ARG load at week 6—a burden that remained persistently high through week 8—but also induced a secondary expansion of resistance breadth, with subtype richness rebounding from roughly 18 at week 6 to 30 by week 8 (Fig. S2c; Fig. S3c). Although type-level PCoA (Fig. S2e) revealed only modest spatial separation—indicating that the predominant resistance classes remained compositionally stable—the quantitative data underscore a clear divergence in risk mitigation: whereas icariin facilitated continuous load reductions without causing structural disruption, copper sulfate exacerbated resistance risks by actively expanding both overall burden and lineage diversity.

Parallel tracking at the MGE level revealed a baseline mobility backbone largely composed of transposases, insertion sequences, integrases, and plasmids, anchored by *tnpA*, *IS91*, and *ISCrsp1* as the dominant subtypes (Fig. S4a; Fig. S3d). During the antibiotic exposure phase, overall MGE loads and compositional profiles remained largely static, closely mirroring the stabilization observed in the ARG network. During the post-withdrawal period, MGE dynamics similarly diverged: the CON group experienced a temporary peak at week 6 before dropping below baseline by week 8 (Fig. S4a); the ICR group achieved a continuous, progressive reduction in mobility elements from week 4 onward while maintaining structural stability (Fig. S4b; Fig. S3e); whereas the CU group exhibited a dramatic accumulation of MGEs by week 6 that was stubbornly sustained through week 8 (Fig. S4c; Fig. S3f). Furthermore, MGE subtype richness in the CU group escalated alarmingly from 3 subtypes at week 4 to 18 subtypes by week 8, a stark deviation from the stabilized, low-richness profile (maintained at ~3 subtypes) observed in the ICR cohort (Fig. S4d). Collectively, the coordinated trajectories of ARGs and MGEs illuminate highly divergent intervention outcomes: natural recovery (CON group) follows a self-limiting "rise-and-fall" pattern; phytochemical intervention (ICR group) actively depresses resistance loads while restricting horizontal mobility potential; and heavy metal supplementation (CU group) exerts a profound co-selective pressure that simultaneously inflates resistance burdens and broadens mobilization capabilities, thereby substantially elevating horizontal gene transfer risks.

### Temporal dynamics of microbial hosts carrying ARGs and MGEs

**Fig. 3:**
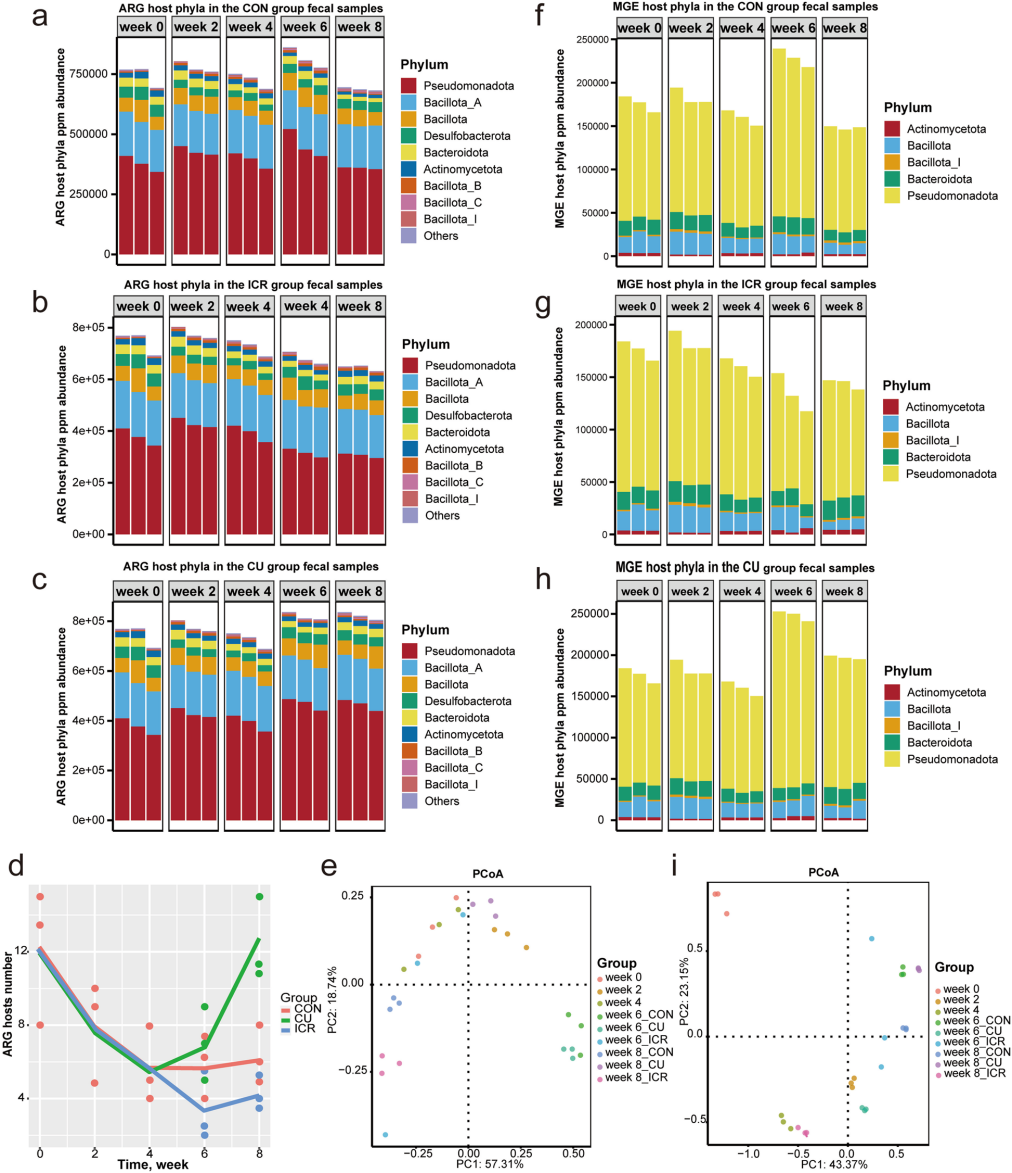
Phylum-level dynamics of antibiotic resistance gene (ARG) and mobile genetic element (MGE) hosts. **a**–**c** Stacked ppm abundances of ARG hosts at the phylum level across time points for the CON, ICR, and CU groups. **f**–**h** Stacked ppm abundances of MGE hosts at the phylum level across time points for the CON, ICR, and CU groups. **d** Line chart of ARG host counts across time points in for three groups. **e **and **i** Principal coordinates analysis (PCoA) plots based on Bray–Curtis distances illustrating differences in the compositional structure of ARG and MGE hosts, respectively. In the stacked-bar plots (**a**–**h**), each bar represents one individual fecal sample (biological replicate), with three replicates (*n* = 3) shown for each group at each time point

To integrate resistance phenotypes with their ecological carriers, metagenomic reads were mapped to putative microbial hosts using the BP-Tracer pipeline, allowing for the comprehensive profiling of host composition, absolute abundance, and structural turnover in relation to ARG and MGE dynamics (Fig. 3; Fig. S5). As anticipated, the temporal trajectories of these resistance hosts closely mirrored the overarching aggregate trends. Taxonomically, the major carriers of ARGs were distributed across Pseudomonadota, Bacillota_A, and Bacillota, while MGE carriage was predominantly concentrated within Pseudomonadota, Actinomycetota, and Bacillota (Fig. 3a and f). Notably, at the genus level, Escherichia emerged as the unequivocally dominant reservoir for both ARGs and MGEs (Fig. S5a and d), underscoring its keystone role in the environmental dissemination network of mobile resistance.

Throughout the eight-week experimental period, dietary interventions primarily modulated absolute carrier loads and genus-level compositions without completely displacing the predominant phyla. During the uniform antibiotic exposure phase (weeks 0–4), the cumulative abundance of carrier hosts remained quantitatively stable: ARG-host loads fluctuated minimally (744,308 ppm at week 0 to 725,317 ppm at week 4), as did MGE-host loads (175,722 to 159,623 ppm) (Fig. 3a and f). However, this numerical stability masked a profound reduction in ARG-host diversity, which plummeted from an average of 12.00 distinct host lineages at week 0 to merely 6.00 by week 4 (Fig. 3d), further evidencing the antibiotic-driven elimination of peripheral carriers and the selective consolidation of a core resistance network.

Post-withdrawal host dynamics varied distinctly by dietary intervention. In the CON group, total host loads exhibited a transient spike at week 6 (ARG hosts: 815,217 ppm; MGE hosts: 228,626 ppm) before receding by week 8 to levels below the pre-withdrawal averages (Fig. 3a, d and f). In contrast, the ICR intervention induced a robust and monotonic depletion of both ARG and MGE hosts from week 4 onwards (ARG hosts down to 645,285 ppm by week 8), tightly coupled with persistently constrained host counts (4.00), signaling a definitive restriction of the resistance dissemination spectrum (Fig. 3b, d and g). Conversely, copper supplementation in the CU group stimulated a sustained overgrowth of resistance-carrying hosts, alongside a near-doubling of host taxonomic richness (from 7.00 at week 4 to 12.67 at week 8), fundamentally expanding the reservoir capable of multidrug transmission (Fig. 3c, d and h). These shifts were corroborated by Bray–Curtis PCoA visualizations, which mapped clear temporal and treatment-specific structural separations for both ARG and MGE carriers (Fig. 3e and i). Ultimately, while maintaining a stable phylum-level architecture, distinct post-withdrawal strategies exert profound modulatory effects on host-level dissemination potential: icariin actively disassembles the pathogen-associated carrier network, whereas heavy metal interventions inadvertently facilitate its expansion.

### Horizontal transfer risk of ARGs in layer hen feces

**Fig. 4:**
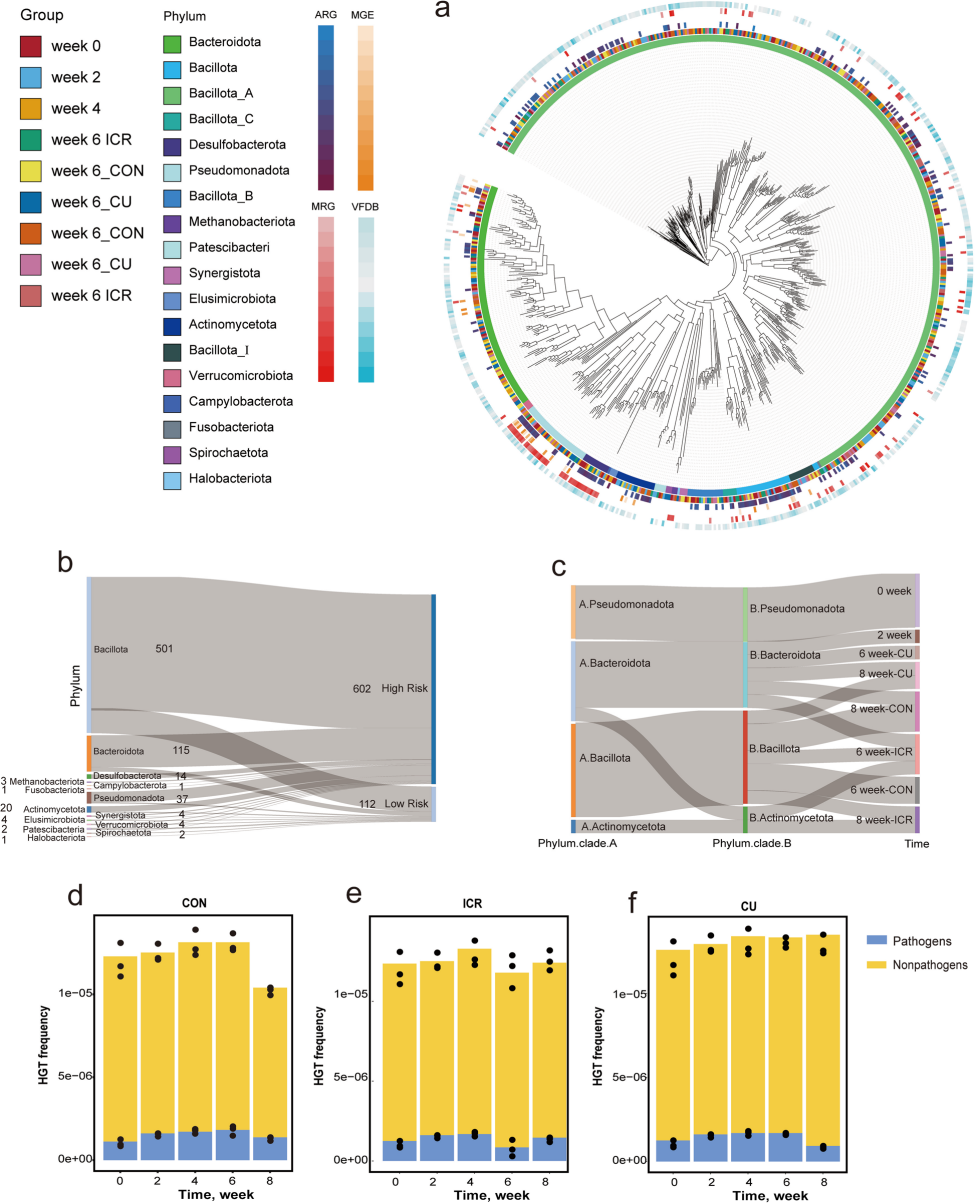
Dominant host taxa and horizontal gene transfer (HGT) patterns associated with antibiotic resistance genes (ARGs) and mobile genetic elements (MGEs) in layer hens. **a** Phylogenetic tree of 709 metagenome-assembled genomes (MAGs) annotated with functional loads, including ARGs, MGEs, metal resistance genes (MRGs), and virulence factors (VFs), highlighting high-risk MAGs. **b** Sankey diagram showing the primary phylum-level composition of MAGs and associated risk levels. **c** Sankey diagram of phyla involved in HGT events and cross-phylum flows (predominantly within-phylum transfer, limited Bacteroidota → Actinomycetota). **d**–**f** Temporal HGT frequencies in pathogenic and nonpathogenic groups for the CON, ICR, and CU groups, respectively. In the stacked-bar plots (**d**–**f**), each bar represents one individual fecal sample (biological replicate), with three replicates (*n* = 3) shown for each group at each time point

To directly quantify dissemination risks, high-quality MAGs were integrated with read-level host attribution, enabling the precise identification of high-risk carrier lineages and the temporal estimation of HGT events (Fig. 4). A total of 709 robust MAGs were recovered, taxonomically aligning with the core structural framework of the fecal microbiome—predominantly deriving from Bacillota_A, Bacteroidota, and Bacillota (Fig. 4a). Within this genomic pool, Pseudomonadota—specifically represented by the genus *Escherichia*—was definitively identified as the principal hub for ARG carriage. Crucially, a specific subset of these MAGs exhibited concurrent genomic carriage of ARGs, MGEs, MRGs, and virulence factors (VFs), characterizing a high-risk multidrug-pathogenicity nexus. Consistent with compositional assessments, icariin intervention successfully diminished the total prevalence of these high-risk MAGs without inducing dramatic structural perturbations to the underlying host spectrum.

Assembly-level HGT tracing revealed that gene transfer events were overwhelmingly confined to within-phylum exchanges—principally originating within Bacillota, followed by Bacteroidota and Pseudomonadota (Fig. 4b)—though sporadic, notable cross-phylum mobilization from Bacteroidota into Actinomycetota was also detected (Fig. 4c). As anticipated, the application of selective drug pressure (weeks 0–4) induced a generalized, systemic elevation in HGT frequencies across both pathogenic and nonpathogenic bacterial communities (Fig. 4d–f).

During the critical post-withdrawal window, dissemination kinetics responded directly to dietary treatments. While HGT proxies in the CON group underwent a transient surge at week 6 before stabilizing, the ICR group achieved a rapid, deep suppression of HGT activity by week 6—effectively dropping below the initial baseline—and maintained these suppressed rates through week 8. Conversely, the CU group exhibited persistently high transfer frequencies at week 6, displaying a delayed attenuation response that corresponds directly with the copper-driven expansion of host ranges and MRG-mediated co-selection. Collectively, these genomic data indicate that while plant-derived icariin swiftly curtails inter-bacterial genetic exchange, inorganic copper promoters extend the temporal window of high-risk horizontal dissemination.

### Key microorganisms driving pathogen-mediated biocontamination in CON group

To mechanically dissect the specific taxa mediating resistance dynamics during both accumulation and natural recovery, we focused on the CON group, integrating absolute load metrics with DESeq2 differential abundance and pathogenic attribution analyses (Fig. S6). By week 4 of antibiotic exposure, the fraction of ARGs and MGEs attributed to pathogenic hosts had increased markedly, whereas carriage by nonpathogens correspondingly contracted (Fig. S6a and b). This proportional shift demonstrates that sub-therapeutic drug pressure effectively clears susceptible commensal background populations while selectively enriching highly competent, pathogen-associated reservoirs, thereby functionally elevating the overall zoonotic risk despite marginal changes in total aggregate loads. Differential abundance analysis (week 4 vs. week 0) captured 14 species demonstrating synchronous, statistically significant increases in both relative abundance and ARG carriage. Intriguingly, all 14 identified species were strictly nonpathogenic, with half originating from Bacillota_A (Fig. S6g), indicating that under acute stress, specific commensal groups act as non-targeted, background "amplifiers" for resistance matrices.

During the natural recovery phase (weeks 4–8), total resistance loads underwent a progressive decline, predominantly driven by the decay of the pathogen compartment, which accounted for a striking 88.24% of total ARG reduction and 57.88% of MGE reduction. A subsequent three-way intersection analysis (capturing species with simultaneous decreases in abundance, ARG carriage, and MGE carriage) identified five pivotal "de-amplification" species. Taxonomically, these converged on *Collinsella*, *Limosilactobacillus*, *Escherichia*, and *Gallibacterium *(Fig. S6m and q). Importantly, the pronounced, synchronized decay of the keystone pathogen *Escherichia* constituted the primary mechanism driving the observed mitigation of high-risk contamination post-withdrawal. Furthermore, 22 nonpathogenic species—principally *Ligilactobacillus*, *Bacteroides*, and *Phocaeicola*—also exhibited coordinated declines in ARG carriage, demonstrating that the post-withdrawal phase relies on both the targeted decay of pathogenic hubs and the widespread, cooperative shedding of auxiliary resistance elements across the commensal background.

### Key microorganisms underlying the reduction of pathogen-mediated biocontamination in ICR group

**Fig. 5:**
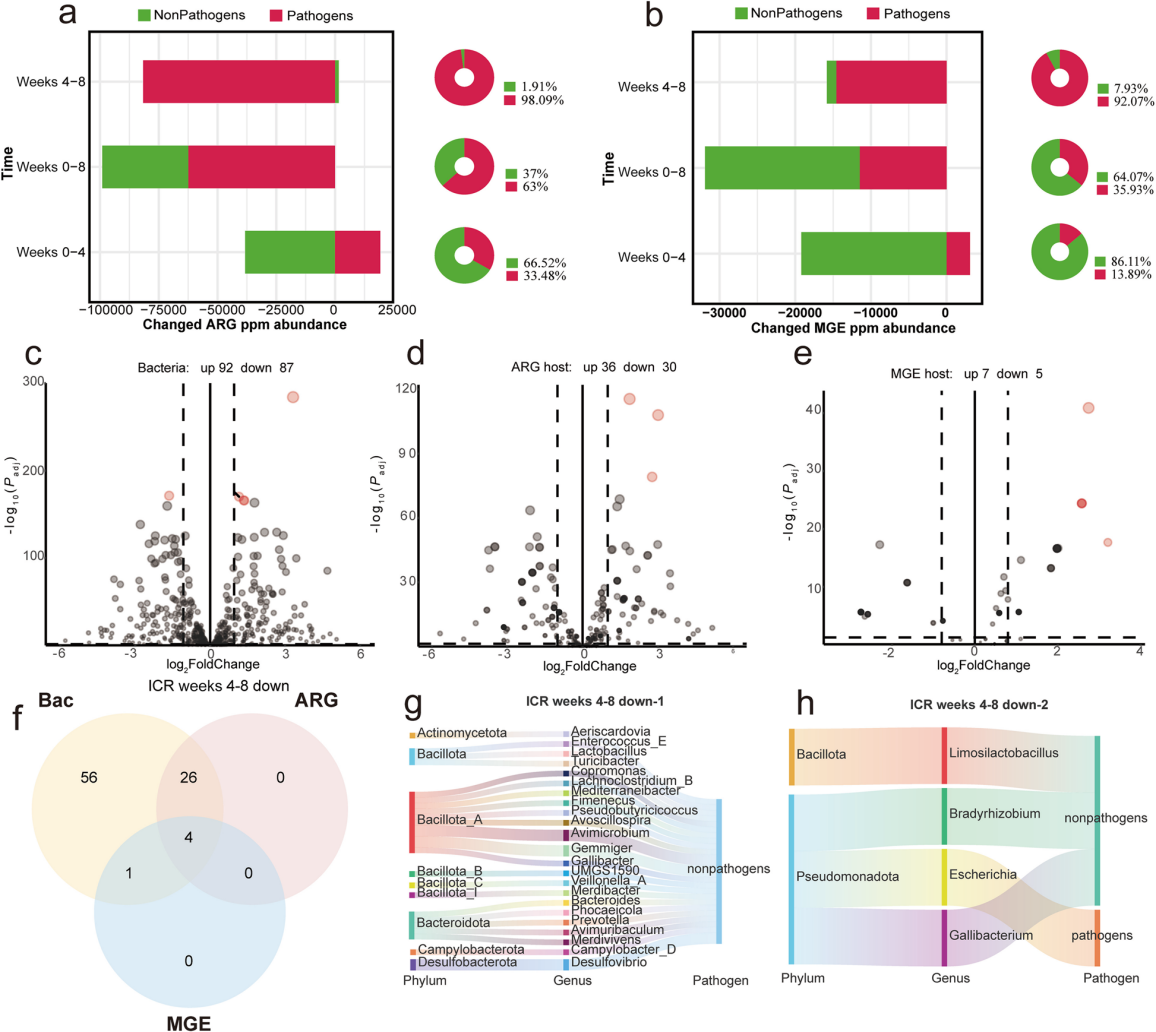
Icariin accelerates decreases in antibiotic resistance genes (ARGs) and mobile genetic elements (MGEs) following discontinuation of low-dose mixed antibiotics. **a **and **b** Changes in ARG and MGE ppm at key time points in the ICR group, with stratified contributions from pathogens and nonpathogens. **c**–**e** Differential abundance analyses (DESeq2) for week 8 vs week 4 following icariin intervention after antibiotic withdrawal (bacterial, ARG-host, and MGE-host abundances). **f** Venn intersection of the three significantly “downregulated” sets (species and ARG and MGE hosts). **g** Taxonomic structure and indicator genera of the two-way intersection (species and ARG hosts). **h** Sankey diagram and taxonomy of the triple-intersection “core species” (*Limosilactobacillus*, *Bradyhizobium*, *Escherichia*, and *Gallibacterium*; *Escherichia* was identified as a pathogen and a key indicator within Pseudomonadota)

To mechanistically evaluate how the phytochemical icariin accelerates "contamination dissipation", an identical multidimensional intersection analysis was applied to the ICR group during the weeks 4–8 intervention window (Fig. 5). Data clearly illustrate that sustained dietary supplementation with icariin yielded highly accelerated reductions in total ARG and MGE densities (Fig. 5a). Notably, contribution partitioning highlighted an almost exclusive reliance on pathogen attenuation; pathogenic communities were responsible for an overwhelming 98.09% of the net ARG reduction and 92.07% of the net MGE reduction. This magnitude of response underscores the potent, targeted inhibitory effects of icariin on highly mobile, pathogen-borne genetic elements, functioning as an exceptionally efficient sequential hazard-reduction strategy.

Intersecting significantly depleted species, depleted ARG carriers, and depleted MGE carriers isolated a precise cluster of "core de-amplification species" responsive to icariin (Fig. 5f). In this study, we define “de-amplification species” as specific microbial taxa that exhibit a synchronous decrease in their overall relative abundance, alongside a marked reduction in their carriage of both ARGs and MGEs. Taxonomically, this cohort converged identically on *Limosilactobacillus*, *Bradyhizobium*, *Escherichia*, and *Gallibacterium*. The presence of *Escherichia* within this core depletion network reaffirms its status as a critical biomarker and indicates that the direct suppression of Pseudomonadota-linked hubs constitutes a primary mechanism driving icariin’s restorative efficacy. Furthermore, 26 distinct commensal species—chiefly distributed among Bacillota_A and Bacteroidota, including indicator genera like *Avimicrobium* and *Gemmiger* (Fig. 5g)—demonstrated robust, simultaneous down-regulation of generalized ARG carriage. This suggests that icariin not only targets specific high-risk pathogenic nodes but also facilitates an accelerated, widespread shedding of metabolic and genetic burdens across the broader nonpathogenic commensal community, achieving holistic resistome remediation.

### Temporal dynamics of pathogen-mediated biocontamination in CU group

**Fig. 6:**
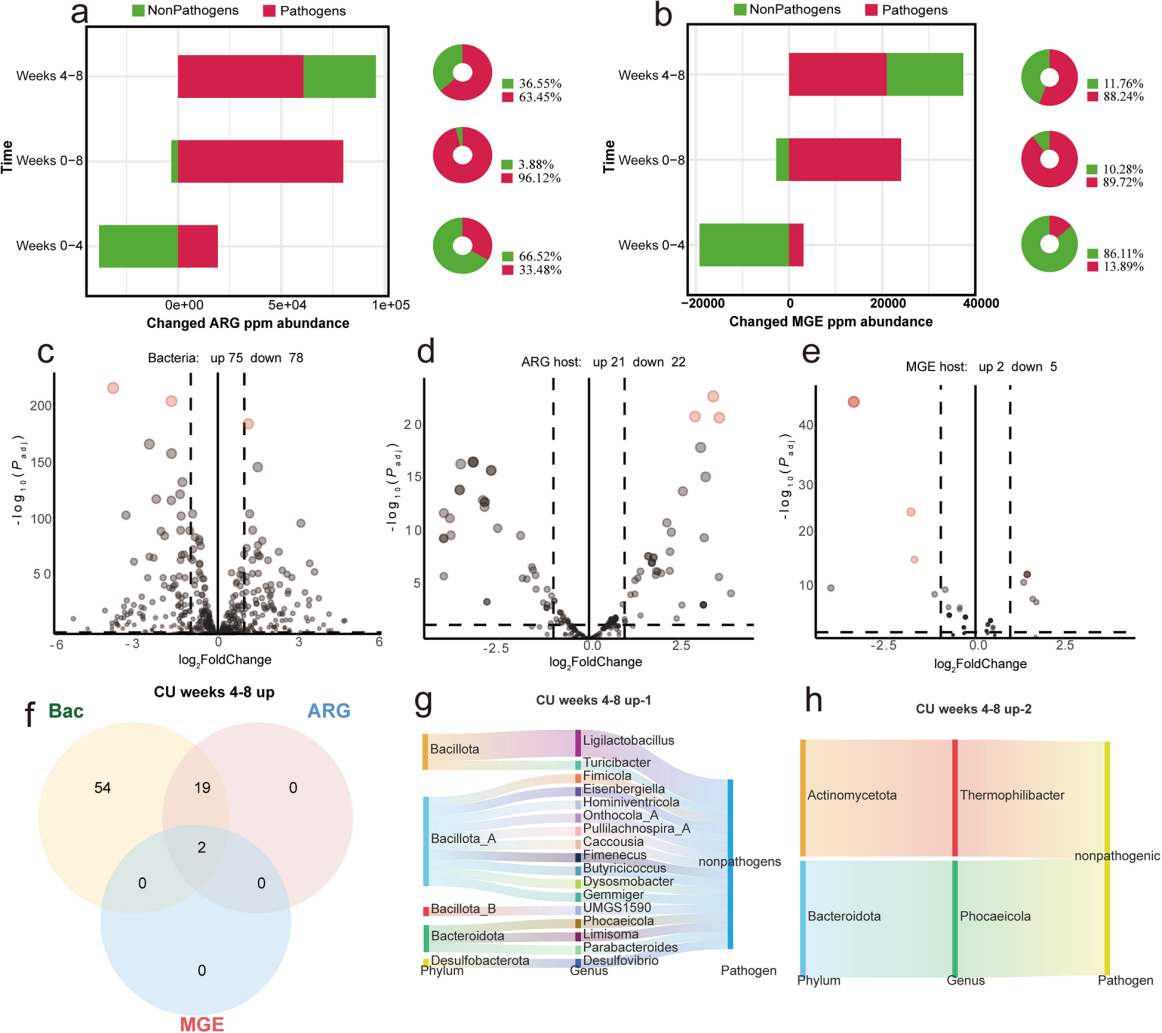
CuSO_4_ increases antibiotic resistance genes (ARGs) and mobile genetic elements (MGEs) after withdrawal of low-dose mixed antibiotics, accelerating the risk of resistance dissemination and biocontamination. **a**, **b** Changes in ARG and MGE ppm at key time points in the CU group, with stratified contributions from pathogens and nonpathogens. **c**–**e** Differential abundance analyses (DESeq2) for week 8 vs week 4 following CuSO_4_ intervention after antibiotic withdrawal (bacterial, ARG-host, and MGE-host abundances). **f** Venn intersection of the three significantly increased sets (species and ARG and MGE hosts). **g** Taxonomic structure and indicator genera for the two-way intersection (species and ARG hosts), dominated by Bacillota_A, Bacillota, and Bacteroidota, with *Ligilactobacillus* as a representative genus. **h** Sankey diagram and taxonomy of triple-intersection “key species” (*Thermophilibacter*, *Phocaeicola*; both nonpathogenic)

Evaluation of the copper-supplemented (CU) group utilized identical integrative metrics to characterize the biological pathways governing secondary resistome expansion post-withdrawal (Fig. 6). In direct contradiction to recovery trends, continuous dietary intervention with 150 mg/kg CuSO_4_ over four weeks precipitated a marked absolute increase in both ARG and MGE burdens (Fig. 6a and b). Stratified attribution mapping confirmed that this surge was disproportionately driven by pathogenic taxa, which were responsible for 63.45% and 88.24% of the net gains in ARGs and MGEs, respectively. These data robustly validate that heavy metal supplementation imposes secondary selective pressures that selectively exacerbate pathogen-mediated dissemination risks.

Intersection of DESeq2 metrics identified a specific cohort of "core amplification species" that concurrently exhibited significant expansions in relative abundance, ARG carriage, and MGE carriage under copper stress. Crucially, the two-way overlap analysis revealed 19 species—exclusively nonpathogenic, largely concentrated within Bacillota_A and Bacillota, and strongly represented by *Ligilactobacillus*—that actively increased their ARG carrier status (Fig. 6g). This broad-based response strongly implicates the nonpathogenic commensal community as cooperative platforms for resistance accumulation via co-selection mechanisms. Consequently, whereas icariin orchestrates a "pathogen-dominated decline", copper sulfate intervention dictates a highly detrimental "pathogen-dominated increase coupled with nonpathogenic co-amplification," fundamentally compromising post-antibiotic ecological restoration.

### Transferable minimal monitoring set anchored on the Pseudomonadota–*Escherichia* axis

**Fig. 7:**
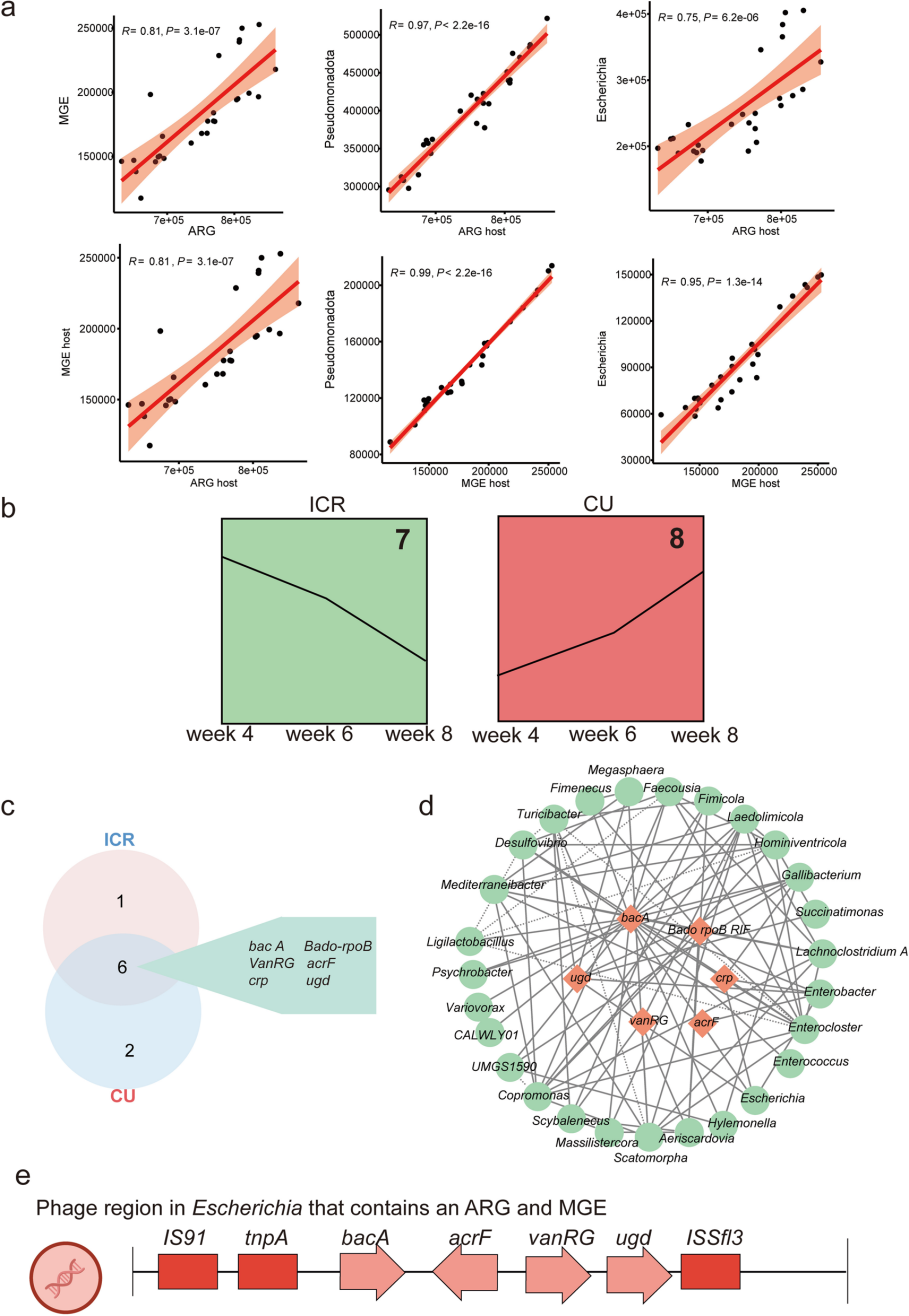
Confirmation of a minimal sentinel set for resistome surveillance via correlation structure, trend concordance, and host–ARG/MGE integration. **a** Linear correlations among total and host‑resolved abundances: total MGEs vs total ARGs; total MGE‑hosts vs total ARG‑hosts; relative abundance of Pseudomonadota vs total ARG‑hosts and total MGE‑hosts; relative abundance of Escherichia vs total ARG‑hosts and total MGE‑hosts. **b** Time‑series concordance of ARG subtype trajectories with total ARGs (ppm): in the ICR group, 7 of the top‑10 ARG subtypes decrease in parallel with total ARGs; in the CU group, 8 of the top‑10 ARG subtypes increase in parallel with total ARGs. **c** Intersection of the ICR and CU concordant sets yields six shared ARG subtypes: *bacA*, *Bado‑rpoB*, *vanRG*, *acrF*, *crp*, and *ugd*. **d** Bipartite network linking the six shared ARG subtypes to their carrier genera (ARG–genus host assignments/co‑occurrence). **e** Minimal sentinel set featuring Escherichia carrying four sentinel ARGs (*bacA*, *acrF*, *vanRG*, *ugd*) and co‑occurring with three MGEs (*IS91*, *tnpA*, *ISSfl3*)

Given the complexity of metagenomic surveillance, we synthesized community, host, and gene-level data to formulate a minimal, operationally viable biomonitoring framework, validated across all treatment conditions and anchored on the Pseudomonadota–*Escherichia* axis alongside a single sentinel resistance gene, *bacA* (Fig. 7). Baseline correlation analyses established profound structural coupling between resistance and mobility networks, revealing highly significant correlations between total MGE and total ARG abundances (*R* = 0.81, *P* < 0.001), as well as between their respective carrier host loads (*R* = 0.81, *P* < 0.001) (Fig. 7a). Critically, the relative abundance of the phylum Pseudomonadota served as a near-perfect proxy for aggregate system risks, exhibiting exceptionally strong linear relationships with both ARG-host loads (*R* = 0.97, *P* < 0.001) and MGE-host loads (*R *= 0.99, *P* < 0.001). Within this taxon, *Escherichia* operated as the keystone statistical node, quantitatively underpinning 24.46% (170,292 ppm) of total ARG carriage and 38.49% (58,475 ppm) of total MGE carriage, solidifying its validity as the primary biological indicator.

Longitudinal profiling of intervention outcomes during the crucial post-withdrawal window (weeks 4–8) further resolved specific molecular targets. By evaluating the trajectory-consistency of the top 10 most abundant ARG subtypes under both restorative (ICR) and exacerbating (CU) conditions, intersection analysis yielded a consensus set of six highly reliable molecular markers: *bacA*, *Bado_rpoB*, *vanRG*, *acrF*, *crp*, and *ugd*, with *bacA* consistently demonstrating supreme absolute abundance across all temporal coordinates (Fig. 7b and c). Biologically, *bacA* functions dually as a bacitracin resistance determinant and a core intrinsic gene of *Escherichia*; its robust statistical performance is therefore inextricably linked to its direct, linear representation of the underlying keystone host population dynamics. Network topologies, constructed via high-stringency Spearman correlations (*ρ* > 0.6), further confirmed *bacA* as the preeminent molecular hub connecting 19 distinct carrier genera (Fig. 7d). While both *Escherichia* and *Enterobacter* exhibited diverse ARG profiles, *Escherichia* uniquely co-localized with three critical MGEs (*IS91*, *tnpA*, *ISSfl3*), an association absent in *Enterobacter* (Fig. 7e).

Based on these integrated findings, we propose a two-tiered, operationally streamlined diagnostic framework. A Tier 1 (Early Warning) alert is activated by isolated threshold exceedances in either *Escherichia* abundance or *bacA* concentration. A Tier 2 (Critical Dissemination Risk) alert is subsequently triggered by either the sustained concurrent elevation of both markers across multiple sampling points or the co-detection of the key MGE (*IS91*, *tnpA*, *ISSfl3*). This minimal configuration successfully distills complex metagenomic architectures into a practical, highly sensitive diagnostic tool optimized for routine agricultural surveillance.

## Discussion

### Subtherapeutic antibiotic-driven ARG accumulation

In AF production systems, ARGs remain inherent elements of the poultry gut microbiome, influenced by environmental inputs, HGT, and co-selection pressures resulting from non-antibiotic stressors [40, 41]. Even after the cessation of antibiotic administration, the AF control group maintained a diverse reservoir of ARGs and MGEs throughout the post-withdrawal period (weeks 5–8). While absolute abundances naturally declined compared to the peak exposure phase, resistance determinants spanning multidrug, MLS, and vancomycin classes remained readily detectable at baseline levels. This trend is consistent with findings in AF swine and broiler studies and highlights the role of environmental and historical inputs in sustaining mobile resistomes [10, 40, 41]. For example, previous research indicates that ARGs such as *tetW* and *aadA* can persist at levels of 10–100 copies per 16S rRNA despite the absence of direct antibiotic [9, 42]. This resilience underscores the role of commensal reservoirs in maintaining resistomes, which can potentially be amplified by feed contaminants or farm biosecurity lapses, as observed in recent European poultry cohorts [43]. Comparatively, our AF baseline demonstrated a greater prevalence of Pseudomonadota-associated ARGs than Bacteroidota. This distribution is similar to human gut studies where Pseudomonadota dominate transient resistance pools, highlighting the importance of implementing context-specific surveillance to differentiate between intrinsic and acquired resistance [44]. Consequently, Pseudomonadota‑linked ARGs require targeted monitoring after antibiotic withdrawal.

The administration of low-dose mixed antibiotics (chlortetracycline 100 g/ton, sulfamethoxazole 100 g/ton, and penicillin 50 g/ton; weeks 0–4) facilitated ARG accumulation through both selective enrichment and co-selection mechanisms, progressively increasing total ARG loads while simultaneously reducing subtype diversity. Initially, overall ARG abundances stabilized; however, by week 4, pathogen-mediated carriage increased sharply, primarily driven by *Escherichia* within the Pseudomonadota phylum. Chlortetracycline, a tetracycline, promoted tetracycline resistance genes through efflux and ribosomal protection mechanisms, consistent with studies exhibiting enhanced plasmid carriage in Enterobacterales under subtherapeutic exposure [45, 46]. Similarly, sulfamethoxazole, a sulfonamide, amplified *sul* gene through target mutations and efflux pump co-expression, as previously observed in *Escherichia* isolates from poultry [47, 48]. Penicillin, a beta-lactam, enriched *bla* gene and other beta-lactamase determinants indirectly by suppressing competing gram-positive taxa, thereby enabling Pseudomonadota expansion and MGE proliferation, similar to the dynamics observed in antibiotic growth promoter studies [49]. This phase-specific accumulation, coupled with the relative enrichment of pathogens, demonstrates how subtherapeutic antibiotic mixtures exacerbate HGT hotspots, potentially increasing zoonotic risks compared to single-antibiotic exposures [50].

### Rebound–decline repair, and legacy ARG residuals following antibiotic withdrawal

Following antibiotic withdrawal, the "rebound–decline" pattern of ARGs/MGEs likely indicates a combination of short-term ecological inertia and the adaptive costs associated with the dissipating selective pressure. Notably, the abrupt removal of bacteriostatic pressure triggers an acute ecological "withdrawal shock", manifesting as the sharp drop in Shannon diversity observed at week 6 in both the CON and CU groups (declining to 7.61 in CU group). During this transient collapse in community evenness, fast-growing opportunistic taxa rapidly proliferate to monopolize newly vacated niches, a well-documented ecological phenomenon driven by competitive release [49, 50]. This localized overgrowth not only skews the community structure but also transiently enhances the dissemination and expansion of resistome elements. In contrast, the sustained administration of icariin effectively buffers this transition, maintaining high community complexity (Shannon index of 8.01 at week 6) and bypassing this vulnerability window. Following this initial shock, as natural microbial competition intensifies, the amplified ARGs undergo a gradual elimination owing to the metabolic burden and regulatory costs of maintaining multidrug resistance, MLS, and vancomycin-related genes. This trajectory is consistent with longitudinal findings following antibiotic withdrawal in poultry and swine [51, 52]. However, host discrimination in the present study revealed that Pseudomonadota, represented by *Escherichia*, dominated during the decline, while the resurgence of nonpathogenic Bacillota likely promoted MGE decay by reducing stress-induced conjugation frequency. This indicates that community rebalancing following antibiotic withdrawal not only reduces ARG modules but also alters the ecological niches of host networks.

From a risk perspective, the simultaneous decline in pathogen-associated ARG/MGE loads indicates a substantial reduction in transmission potential and health risks. However, the detection of residual "legacy ARGs" suggests the persistence of environmental and chromosomal gene pools, making complete ecological restoration unlikely with short-term (4-week) withdrawal period. This finding underscores the importance of phased withdrawal strategies and precise monitoring. Using the Pseudomonadota–*Escherichia* axis as the minimum monitoring unit enables the identification of key actionable ARG/MGE variants during the recovery phase. Additionally, if the objective is to accelerate resistance elimination and reduce the risk of reintroduction, withdrawal should be combined with complementary strategies such as environmental source control (bedding/water system), microecological interventions (targeted probiotics/competitive exclusion), or conjugation inhibition [53, 54]. This study has certain limitations, primarily the relatively short observation period and the exclusive focus on fecal samples. Further investigation is required to assess gastrointestinal segmental differences and the effects of environmental re‑inoculation. Future research should extend the duration of time series analyses, include environmental sampling, and validate fitness costs and plasmid stability using in vitro with functional assays. Ultimately, the development of a predictive “withdrawal–rebalance–residue” model may help inform tiered risk management strategies.

### Icariin-augmented resistome repair: mechanisms and host ecology

Natural phytochemicals such as icariin accelerated resistome repair at a rate that exceeded antibiotic withdrawal alone. Here, we define “resistome repair” as the ecological process by which the overall burden, diversity, and dissemination potential of ARGs and MGEs within a microbial community are restored toward baseline or low-risk levels after the cessation of selective pressure. This is demonstrated by sustained reductions in ARG/MGE burdens and inhibited HGT from weeks 4–8, with disproportionate effects on pathogen-associated elements. Compared to the control group, icariin intervention yielded a more rapid and substantial decline, particularly within pathogen-linked resistance modules. These findings suggest that icariin is a lower‑hazard alternative to inorganic promoters in post‑antibiotic layer production. By attenuating pathogen‑linked ARG/MGE modules at the source without introducing persistent metal loads, icariin may help reduce the downstream environmental resistome burden associated with manure management and land application. Mechanistically, the targeted depletion of Pseudomonadota (notably *Enterobacteria* such as *Escherichia*) under icariin treatment likely operates through a synergistic interplay of direct anti-virulence action and indirect ecological competitive exclusion, rather than traditional bactericidal killing. Directly, while high concentrations of flavonoids can perturb the Gram-negative outer membrane, the dietary inclusion level applied in this study (200 mg/kg) predominantly exerts sub-inhibitory, "anti-virulence" effects [12, 55]. Specifically, it inhibits quorum sensing to impair biofilm maturation and block pathogen adhesion. This mechanism strips Pseudomonadota of their colonization advantages and limits MGE mobilization without triggering strong survival selective pressures [56]. Furthermore, targeted interference with virulence systems dissociates multidrug resistance from host fitness benefits, facilitating the spontaneous loss of costly ARG-bearing plasmids [57]. Ecologically, icariin acts as a prebiotic-like catalyst that promotes the rapid proliferation of competitive commensals, consistent with its known capacity to reshape avian gut microbiota [18, 19]. This is strongly supported by our temporal data, where icariin sustained *Phocaeicola* and distinctly elevated *Akkermansia* abundance to 6.80% during the recovery phase. The expansion of these beneficial taxa remodels the intestinal microenvironment—likely via short-chain fatty acid production and subsequent luminal pH reduction—which systematically and spatially outcompetes the remaining Pseudomonadota populations. Together, these multi-pronged, low-selection-pressure mechanisms concurrently alleviate host oxidative stress to suppress RecA–LexA-mediated SOS responses [56], thereby deeply reducing both transfer opportunity and plasmid stability. Host-resolved analyses revealed a more restricted host range, particularly within the Pseudomonadota phylum. This aligns aligning the mechanism with the outcome: transfer-active, pathogen-linked modules were selectively attenuated rather than broadly depleted across the community. Relative to the natural repair in the control group, icariin produced rapid and substantial reductions, with pathogens accounting for 98.09% of ARG and 92.07% of MGE decreases; *Escherichia* was identified as a key de-amplification node exhibiting simultaneous decreases in abundance and ARG/MGE carriage. This pathogen-focused attenuation indicates a targeted interruption of high-risk transfer pathways and a contraction of the mobile resistome’s ecological niche, thereby reducing the dissemination potential without introducing new selection pressures—features which are desirable for hazard minimization in production systems [58]. However, the continued presence of “legacy” ARGs underscores incomplete repair within the current observation period and signals the necessity for prolonged monitoring to determine the recovery half-life and steady states. To directly validate these mechanisms, future in vivo and in vitro studies should empirically test quorum sensing inhibition, biofilm formation, SOS activity, conjugation frequencies, and plasmid stability, ideally complemented by large-scale field trials [59].

### Copper-driven co-selection expands mobile resistomes in poultry systems

In contrast to icariin, CuSO_4_ supplementation intensified the expansion of the resistome compared to the withdrawal baselines, increasing total ARG loads and widening MGE host connectivity with a pronounced pathogen bias. Together, these patterns indicate that CuSO₄ sustains and diversifies fecal mobile resistomes through metal‑driven co‑selection even after antibiotic withdrawal. These copper‑linked ARG/MRG clusters in feces are likely to be transferred to manure‑amended soils and receiving waters, where they may increase the ecotoxicological burden by stabilizing multi‑resistant environmental reservoirs and altering key biogeochemical processes. The genomic data provided clear ecological signals of copper-driven co-selection. MRGs co-located with ARGs on plasmids and integrative and conjugative elements were enriched, facilitating multidrug phenotypes through shared efflux and membrane homeostasis systems [60]. Under Cu^2+^ stress, oxidative damage and RecA–LexA–mediated SOS activation likely increased conjugation/transposition rates and stabilized integrative conjugative elements in Enterobacterales (notably *Escherichia*), thereby increasing transfer competence and the retention of costly resistance cassettes [61]. These mechanisms are consistent with observations in heavy metal–polluted soils where copper gradients correlate with ARG proliferation, thereby supporting a conserved co-selection axis extending from environmental matrices to animal gastrointestinal tracts [62]. Shifts in the microbiome led to an increase in Pseudomonadota donors and a decrease in commensals, thereby broadening host associations. The delayed decline in HGT proxies following CuSO_4_ administration indicates an extended period of transfer potential, suggesting that metal-based promoters can sustain mobile resistome amplification even in antibiotic-free production processes. This persistence potentially increases zoonotic spillover risk by maintaining pathogen-centered hubs with high mobilization capacity and by disseminating genetic material into environmental reservoirs through manure streams [63]. From a hazard-management perspective, copper supplementation compromises post-withdrawal repair dynamics by preserving high-risk MGEs and co-selected ARG/MRG clusters. Mitigation strategies should therefore shift toward a multifaceted approach. Primary interventions must include enforcing strict upper-bound dosing or entirely avoiding copper additives, substituting them with non-metal growth promoters that circumvent co-selection pathways. Furthermore, these dietary shifts should be coupled with robust source control and targeted manure treatments—such as applying metal sorbents or advanced redox-controlled composting—to achieve simultaneous metal and ARG removal. To validate the efficacy of these measures, routine environmental surveillance must specifically track the Pseudomonadota–*Escherichia* axis and plasmid-borne MRG–ARG modules as leading indicators of horizontal transfer risk. These findings demonstrate that post-withdrawal promoter selection critically influences resistome trajectories: icariin consistently accelerates remediation by reducing ARG/MGE burdens and HGT, particularly within pathogen-linked Pseudomonadota–*Escherichia* networks, whereas CuSO_4_ increases and prolongs mobile resistome expansion through co-selection with MRGs and sustained transfer potential. In conclusion, icariin provides a safer, mechanism-aligned alternative to metal-based promoters, whereas CuSO_4_ should be constrained owing to its risk-amplifying profile. Integrating targeted surveillance with microbiome‑supportive interventions and evidence‑based policy will ultimately help mitigate resistance hazards and align with One Health goals.

### A transferable minimal sentinel set and decision thresholds for field implementation

Translating high-dimensiona longitudinal metagenomic data into practical farm management requires simple, source-oriented indicators that capture key dimensions of resistome hazard, including abundance, mobility, and host attribution, without relying on cost-prohibitive comprehensive sequencing at every time point [64–66]. To address this bottleneck, we propose a streamlined surveillance framework anchored in the Pseudomonadota–*Escherichia* axis, utilizing *bacA* as a core single-gene marker. This approach converts complex microbial dynamics into actionable signals by integrating host identification, resistance gene prioritization, and horizontal transfer markers into a unified metric. Consistent with reports that Pseudomonadota, and *Escherichia* in particular, often concentrate mobile ARGs and metal resistance determinants in complex aquatic and waste matrices [67–69], *Escherichia* emerged as a sentinel taxon strongly associated with overall resistance risks in our study, while *bacA* was the highest‑abundance hub subtype across treatments and time points. Simultaneously, *bacA* consistently presented as the highest-abundance hub subtype across all treatments and time points. Biologically, while the detection of *bacA* is largely redundant to identifying the *Escherichia* host itself, as it functions simultaneously as an intrinsic core gene of this taxon and a bacitracin resistance determinant. From an operational surveillance perspective, this inherent redundancy translates into a distinct methodological advantage. Specifically, *bacA* provides a highly stable and quantifiable molecular target that reliably proxies the biomass of the high-risk host reservoir. Building on this quantitative anchor, we conceptualize a two-tier alert system with structured decision thresholds. Tier‑1 (early attention) is triggered when either the *bacA* or *Escherichia* indicator exceeds its threshold at any single time point; Tier‑2 (high risk) is activated when both exceed thresholds for ≥ 2 consecutive time points and associated MGEs (such as *IS91*, *tnpA*, *ISSfl3*) are co‑detected—reflecting an active and concurrent elevation of load, host competency, and mobility potential. Crucially, while *bacA* flags the baseline host burden, the actual risk of horizontal gene transfer is independently captured by the co-occurrence of these MGEs, thereby reflecting a genuine and concurrent elevation of the overall resistance load, host competency, and mobility potential. This graded structure mirrors ecotoxicological risk assessment frameworks, in which lower‑tier exceedances trigger heightened surveillance, whereas persistent, multi‑parameter exceedances prompt immediate intervention and mitigation [70, 71]. In practice, this framework enables rapid PCR-based quantification of *Escherichia* and *bacA* for routine screening, reserving expensive reflex metagenomics exclusively for Tier‑2 events to profile MGE context and host expansion. This reduces monitoring cost while preserving sensitivity to hazard escalation and provides explicit triggers for source control (copper dose reduction, bedding/water remediation) and microbiome‑supportive interventions. By aligning thresholds with actionable responses, the framework conforms to risk‑centric decision‑making in One Health settings and is portable across farms and time windows [72, 73]. Although derived from on‑host fecal data, the *Escherichia*–*bacA* sentinel is directly translatable to manure, soil and water samples, where it may serve as a cost‑effective biomarker panel for environmental surveillance of poultry‑derived resistome hazards and for tracking Cu‑linked ARG/MRG clusters as they move from feces into manure‑amended soils and receiving waters. Consequently, the minimal sentinel set provides a practical bridge between high‑resolution metagenomic insights and routine environmental monitoring, enabling regulators and producers to detect and respond to emerging resistome risks before they become entrenched in environmental reservoirs [74, 75].

### Conceptual contributions and implications for one health

The conceptual framework of this study expands traditional antimicrobial resistance research by shifting the analytical focus toward the critical post-antibiotic withdrawal period. Continuous low-dose antibiotic exposure in intensive poultry production fundamentally destabilizes the commensal microbiota, establishing an artificially inflated baseline of mobile resistance. Our longitudinal tracking results indicate that natural recovery following antibiotic cessation remains a slow and often incomplete process [74]. The remaining resistance genes are highly susceptible to secondary expansion during this vulnerable ecological window. Addressing this persistent challenge requires active interventions that can accelerate the clearance of resistance elements before resistant pathogens spread into the surrounding environment [62].

To meet this practical need for active mitigation, the targeted application of phytochemicals offers a sustainable strategy for facilitating ecological restoration. Traditional alternative additives are often selected primarily for their ability to maintain animal growth, sometimes overlooking their environmental impacts. Indeed, our comparative data show that metal-based additives such as copper sulfate can actually maintain or even expand the mobile resistome through the co-selection of heavy metal and antibiotic resistance genes [75]. By contrast, natural compounds like icariin exert a targeted ecological pressure that selectively limits the bacterial hosts carrying resistance genes [18, 19]. By suppressing pathogen-driven HGT without imposing heavy biocidal selection, icariin fundamentally recalibrates the resistome trajectory within farm settings. This dual functionality—promoting physiological recovery while actively dismantling resistance networks—ultimately establishes a biologically sound foundation for the aforementioned One Health surveillance strategies [76, 77].

## Conclusion

In this study, we first established a standardized high-ARG fecal microbiota model in layer hens with a low-dose in-feed antibiotic mixture to elucidate how icariin, a botanical compound previously proven by our team to improve poultry intestinal and immune health, can alleviate the impacts on the fecal resistome after antibiotic withdrawal. By coupling these novel microbiological insights with our prior physiological findings, we demonstrate that icariin can serve as a comprehensive and ecologically safe feed additive. In addition, we proposed utilizing poultry manure as an early warning environment for monitoring and predicting the proliferation and deterioration of ARGs. In particular, we uncovered that a significant correlation exists between host dynamics and mobile resistance networks. The abundance of *Escherichia*, which harbors a prevalent *bacA* subtype, is strongly linked to the loads of mobile ARGs and MGEs. This robust connection positions the *Escherichia*-*bacA* axis as a straightforward and transferable tool for monitoring antibiotic resistance in poultry.

## Supplementary Information

Additional file 1: Fig. S1. Temporal dynamics of the pathogenic subcommunity in layer hens under different growth-promotion strategies. (a–c) Pathogen species composition across time points in CON, ICR, and CU groups, respectively. (d) Line chart showing changes in the Shannon diversity index for pathogen species. (e) Principal coordinates analysis (PCoA) based on Bray-Curtis distances illustrating the overall structure of the pathogenic community. Overall temporal trends mirrored those of the total microbiome: during post-withdrawal recovery, pathogen communities exhibited distinct structural separation among treatments, with greater dispersion between the ICR and CU groups at week 8, suggesting that different promoters differentially influenced colonization patterns and competitive dynamics among potentially pathogenic taxa. Fig. S2. Temporal dynamics of antibiotic resistance gene (ARG) types in layer hens under different growth-promotion strategies. a-c) Stacked ppm abundance of ARG types across time points for the CON, ICR, and CU groups, respectively. d) Line chart of ARG subtype counts across time points for the three groups. e) Principal coordinates analysis (PCoA) of ARG compositional structure based on Bray-Curtis distances. Fig. S3. Temporal dynamics of mobile genetic element (MGE) types. a-c) Stacked ppm abundance of MGE types across time points for the CON, ICR, and CU groups, respectively. d) Line chart of MGE subtype counts across time points for the CON, ICR, and CU groups. e) Principal coordinates analysis (PCoA) of MGE type composition across time points for CON, ICR, and CU groups. Fig. S4. Temporal dynamics of antibiotic resistance gene (ARG) and mobile genetic element (MGE) subtypes. (a–c) Stacked bar plots showing the abundance (parts per million, ppm) of ARG subtypes across time points in the CON, ICR, and CU groups, respectively. (d-f) Stacked bar plots showing the abundance (ppm) of MGE subtypes across time points in the same three groups. Fig. S5. Genus-level dynamics and correlations of antibiotic resistance gene (ARG) and mobile genetic element (MGE) hosts. (a–c) Stacked bar plots showing the abundance (ppm) of ARG-host genera in the CON, ICR, and CU groups, respectively. (d-f) Stacked bar plots showing the abundance (ppm) of MGE-host genera in the same groups. Fig. S6. Key microorganisms associated with pathogen-mediated biocontamination before and after low-dose mixed antibiotics. a) Changes in antibiotic resistance gene (ARG) ppm at key time points in the CON group (stratified by pathogens vs. nonpathogens). b) Changes in mobile genetic element (MGE) ppm at key time points in the CON group (stratified by pathogens vs. nonpathogens). c-e) Differential abundance analyses (DESeq2) for week 4 vs. week 0 across the microbiome, ARG hosts, and MGE hosts. f) Venn intersection of species/ARG hosts/MGE hosts with significant increases at week 4. g) Major taxonomic attributes of key species with significant increases at week 4. h-j) Differential abundance analyses (DESeq2) for week 8 vs. week 4 across the microbiome, ARG hosts, and MGE hosts. q) Venn intersection of species/ARG hosts/MGE hosts with significant decreases at week 8 vs. week 4. l) Taxonomic structure of the intersection “species significantly decreased and ARG host significantly decreased” at week 8 vs. week 4. m) Taxonomic structure of the triple intersection “species significantly decreased and ARG host significantly decreased and MGE host significantly decreased” at week 8 vs. week 4.

## Data Availability

All sequencing data generated in this study have been deposited in the NCBI Sequence Read Archive under BioProject accession number PRJNA1345554. Additional processed data supporting the findings of this work are available from the corresponding author upon reasonable request.

## Abbreviations

AF: Antibiotic-free
ARGs: Antibiotic resistance genes
CuSO₄: Copper sulfate
HGT: Horizontal gene transfer
HPLC: High‑performance liquid chromatography
MAGs: Metagenome-assembled genomes
MGEs: Mobile genetic elements
MLS: Macrolide-lincosamide-streptogramin
MRGs: Metal resistance genes
ORFs: Open reading frames
PCoA: Principal coordinates analysis
SARG: Structured Antibiotic Resistance Gene
VF: Virulence factor
VFDB: Virulence Factor Database
