# Supplementary material for: Icariin shapes post-withdrawal fecal resistome dynamics in layer hens
Source: J Anim Sci Biotechnol. 2026 Jun 4;17:109. doi: 10.1186/s40104-026-01433-8 (PMC13234970; doi:10.1186/s40104-026-01433-8)
Supplement: Supplementary file 1 — Additional file 1: Fig. S1. Temporal dynamics of the pathogenic subcommunity in layer hens under different growth-promotion strategies. (a–c) Pathogen species composition across time points in CON, ICR, and CU groups, respectively. (d) Line chart showing changes in the Shannon diversity index for pathogen species. (e) Principal coordinates analysis (PCoA) based on Bray-Curtis distances illustrating the overall structure of the pathogenic community. Overall temporal trends mirrored those of the total microbiome: during post-withdrawal recovery, pathogen communities exhibited distinct structural separation among treatments, with greater dispersion between the ICR and CU groups at week 8, suggesting that different promoters differentially influenced colonization patterns and competitive dynamics among potentially pathogenic taxa. Fig. S2. Temporal dynamics of antibiotic resistance gene (ARG) types in layer hens under different growth-promotion strategies. a-c) Stacked ppm abundance of ARG types across time points for the CON, ICR, and CU groups, respectively. d) Line chart of ARG subtype counts across time points for the three groups. e) Principal coordinates analysis (PCoA) of ARG compositional structure based on Bray-Curtis distances. Fig. S3. Temporal dynamics of mobile genetic element (MGE) types. a-c) Stacked ppm abundance of MGE types across time points for the CON, ICR, and CU groups, respectively. d) Line chart of MGE subtype counts across time points for the CON, ICR, and CU groups. e) Principal coordinates analysis (PCoA) of MGE type composition across time points for CON, ICR, and CU groups. Fig. S4. Temporal dynamics of antibiotic resistance gene (ARG) and mobile genetic element (MGE) subtypes. (a–c) Stacked bar plots showing the abundance (parts per million, ppm) of ARG subtypes across time points in the CON, ICR, and CU groups, respectively. (d-f) Stacked bar plots showing the abundance (ppm) of MGE subtypes across time points in the same three groups. Fi [file 40104_2026_1433_MOESM1_ESM.docx]

**Supplementary Information**


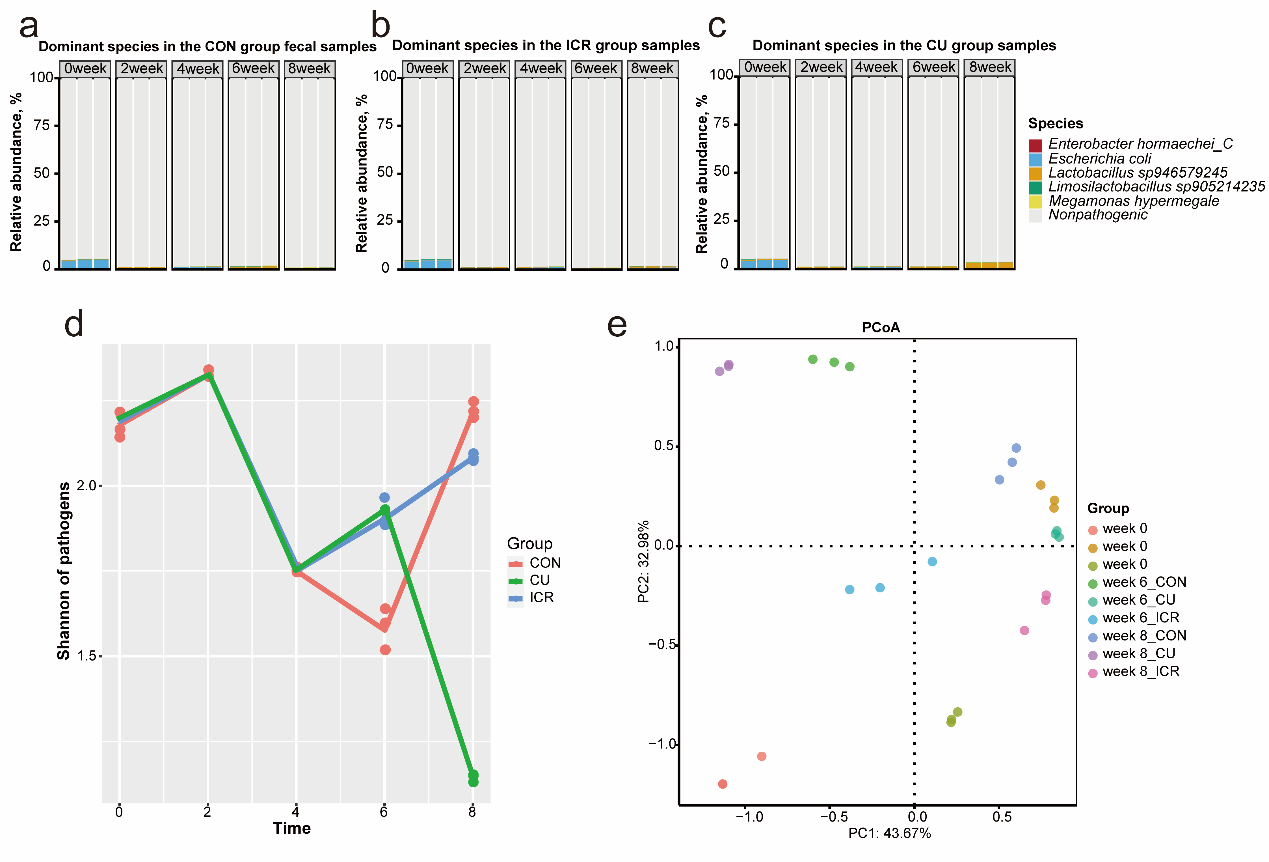


**Fig. S1.** Temporal dynamics of the pathogenic subcommunity in layer hens under different growth-promotion strategies. (a–c) Pathogen species composition across time points in CON, ICR, and CU groups, respectively. (d) Line chart showing changes in the Shannon diversity index for pathogen species. (e) Principal coordinates analysis (PCoA) based on Bray-Curtis distances illustrating the overall structure of the pathogenic community. Overall temporal trends mirrored those of the total microbiome: during post-withdrawal recovery, pathogen communities exhibited distinct structural separation among treatments, with greater dispersion between the ICR and CU groups at week 8, suggesting that different promoters differentially influenced colonization patterns and competitive dynamics among potentially pathogenic taxa. In the stacked-bar plots (a–c), each bar represents one individual fecal sample (biological replicate), with three replicates (n=3) shown for each group at each time point. In the stacked-bar plots (a-c), each bar represents one individual fecal sample (biological replicate), with three replicates (n=3) shown for each group at each time point.


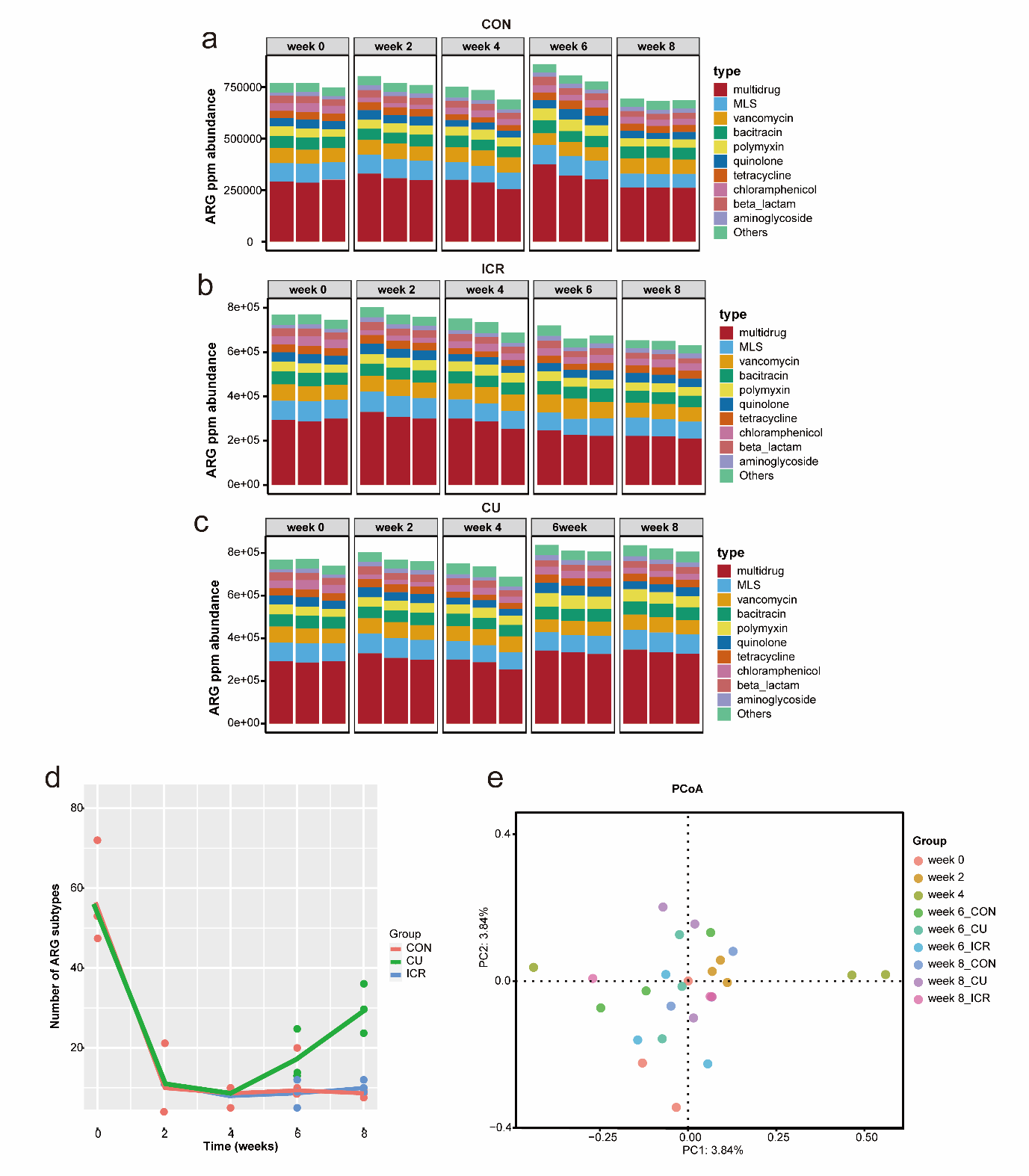


**Fig. S2.** Temporal dynamics of antibiotic resistance gene (ARG) types in layer hens under different growth-promotion strategies. a–c) Stacked ppm abundance of ARG types across time points for the CON, ICR, and CU groups, respectively. d) Line chart of ARG subtype counts across time points for the three groups. e) Principal coordinates analysis (PCoA) of ARG compositional structure based on Bray–Curtis distances. In the stacked-bar plots (a-c), each bar represents one individual fecal sample (biological replicate), with three replicates (n=3) shown for each group at each time point.


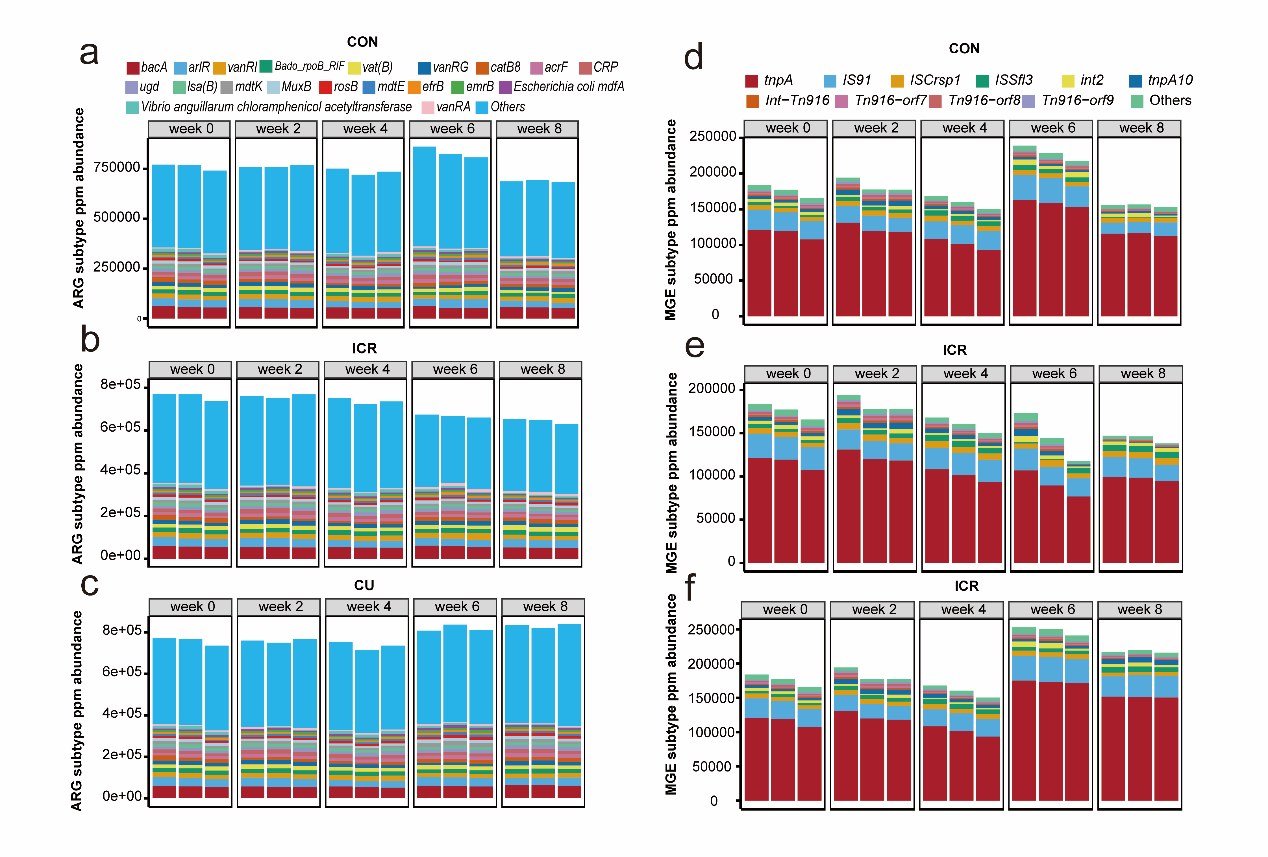


**Fig. S3.** Temporal dynamics of mobile genetic element (MGE) types. a–c) Stacked ppm abundance of MGE types across time points for the CON, ICR, and CU groups, respectively. d) Line chart of MGE subtype counts across time points for the CON, ICR, and CU groups. e) Principal coordinates analysis (PCoA) of MGE type composition across time points for CON, ICR, and CU groups. In the stacked-bar plots (a-f), each bar represents one individual fecal sample (biological replicate), with three replicates (n=3) shown for each group at each time point.


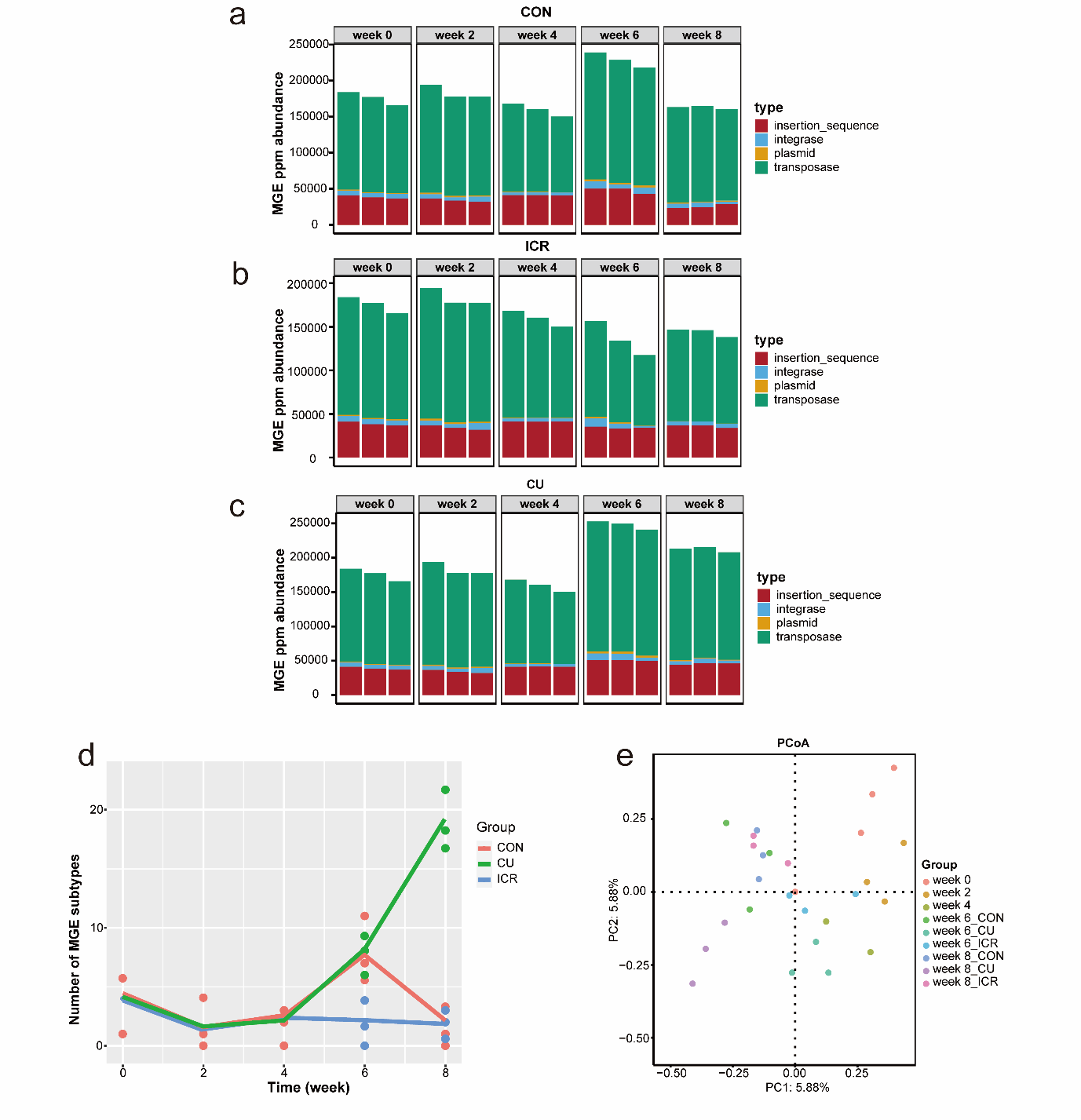


**Fig. S4.** Temporal dynamics of antibiotic resistance gene (ARG) and mobile genetic element (MGE) subtypes. (a–c) Stacked bar plots showing the abundance (parts per million, ppm) of ARG subtypes across time points in the CON, ICR, and CU groups, respectively. (d–f) Stacked bar plots showing the abundance (ppm) of MGE subtypes across time points in the same three groups. In the stacked-bar plots (a-c), each bar represents one individual fecal sample (biological replicate), with three replicates (n=3) shown for each group at each time point.


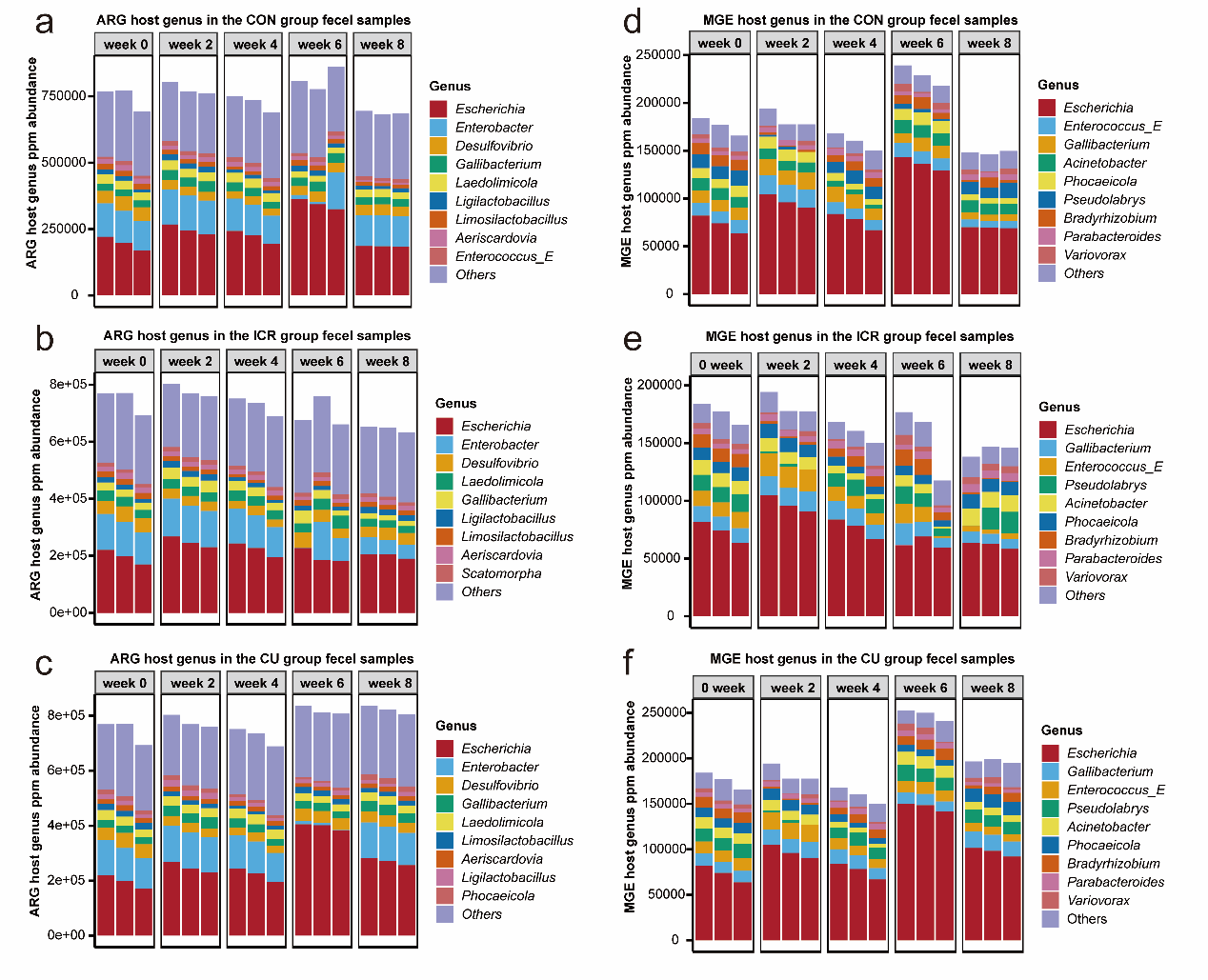


**Fig. S5.** Genus-level dynamics and correlations of antibiotic resistance gene (ARG) and mobile genetic element (MGE) hosts. (a–c) Stacked bar plots showing the abundance (ppm) of ARG-host genera in the CON, ICR, and CU groups, respectively. (d–f) Stacked bar plots showing the abundance (ppm) of MGE-host genera in the same groups. In the stacked-bar plots (a-f), each bar represents one individual fecal sample (biological replicate), with three replicates (n=3) shown for each group at each time point.


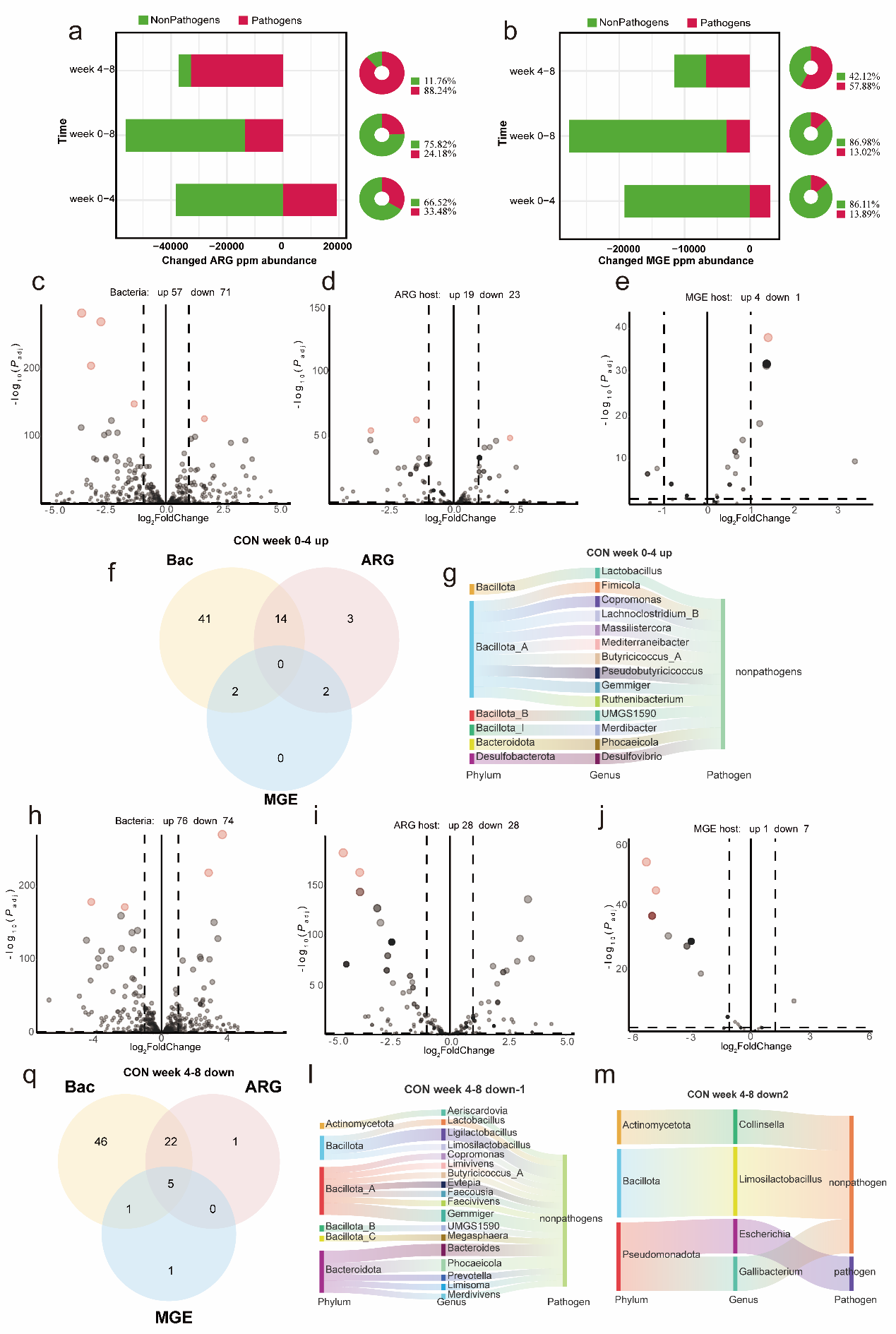


**Fig. S6.** Key microorganisms associated with pathogen-mediated biocontamination before and after low-dose mixed antibiotics. a) Changes in antibiotic resistance gene (ARG) ppm at key time points in the CON group (stratified by pathogens vs. nonpathogens). b) Changes in mobile genetic element (MGE) ppm at key time points in the CON group (stratified by pathogens vs. nonpathogens). c–e) Differential abundance analyses (DESeq2) for week 4 vs. week 0 across the microbiome, ARG hosts, and MGE hosts. f) Venn intersection of species/ARG hosts/MGE hosts with significant increases at week 4. g) Major taxonomic attributes of key species with significant increases at week 4. h–j) Differential abundance analyses (DESeq2) for week 8 vs. week 4 across the microbiome, ARG hosts, and MGE hosts. q) Venn intersection of species/ARG hosts/MGE hosts with significant decreases at week 8 vs. week 4. l) Taxonomic structure of the intersection “species significantly decreased and ARG host significantly decreased” at week 8 vs. week 4. m) Taxonomic structure of the triple intersection “species significantly decreased and ARG host significantly decreased and MGE host significantly decreased” at week 8 vs. week 4.

**Table S1**. Ingredients and chemical composition of the basal diet (dry matter basis).

| **Item** | **Content** |
| --- | --- |
| **Ingredient, %** |  |
| Corn | 54.5 |
| Wheat | 10.0 |
| Soybean meal | 16.85 |
| Rapeseed meal | 4.0 |
| Rice bran meal | 3.5 |
| Corn protein powder | 0.60 |
| Soybean oil | 0.25 |
| Limestone | 8.4 |
| Dicalcium phosphate | 0.62 |
| Lysine-HCl (70%) | 0.15 |
| dl-Methionine | 0.09 |
| Salt (NaCl) | 0.4 |
| Choline chloride | 0.1 |
| Vitamin premix^1^ | 0.03 |
| Mineral premix^2^ | 0.5 |
| Phyzyme XP 5000G | 0.01 |
| Total | 100 |
| **Chemical composition^3^, %** |  |
| ME, MJ/kg | 11.0 |
| Crude protein | 15.3 |
| Calcium | 3.8 |
| Available phosphorus | 0.35 |
| Lysine | 0.75 |
| Methionine | 0.35 |
| Methionine + Cystine | 0.54 |
| Threonine | 0.58 |
| Tryptophan | 0.19 |

^1^Provided per kilogram of diet: retinyl acetate, 3.1 mg; cholecalciferol, 0.0375 mg; DL-α-tocopheryl acetate, 7.5 mg; thiamin, 0.6 mg; riboflavin, 4.8 mg; pyridoxine hydrochloride, 1.5 mg; cyanocobalamin, 0.009 mg; calcium-D-pantothenate, 7.5 mg; folic acid, 0.15 mg; niacin, 20 mg.

^2^Provided per kilogram of diet: copper (CuSO_4_·5H_2_O), 6 mg; iron (FeSO_4_·H_2_O), 60 mg; zinc (ZnSO_4_·H_2_O), 80 mg; manganese (MnSO4·H2O), 60 mg; selenium (NaSeO_3_), 0.3 mg; iodine (KI), 0.35 mg.

^3^The value of crude protein was analyzed and the value of metabolizable energy (ME) was calculated, others were calculated values.
